# Identification of Novel High-Frequency DNA Methylation Changes in Breast Cancer

**DOI:** 10.1371/journal.pone.0001314

**Published:** 2007-12-19

**Authors:** Jared M. Ordway, Muhammad A. Budiman, Yulia Korshunova, Rebecca K. Maloney, Joseph A. Bedell, Robert W. Citek, Blaire Bacher, Seth Peterson, Tracy Rohlfing, Jacqueline Hall, Robert Brown, Nathan Lakey, Rebecca W. Doerge, Robert A. Martienssen, Jorge Leon, John D. McPherson, Jeffrey A. Jeddeloh

**Affiliations:** 1 Orion Genomics, St. Louis, Missouri, United States of America; 2 Centre for Bioinformatics, McGill University, Montreal, Quebec, Canada; 3 Imperial College, Hammersmith Hospital, London, United Kingdom; 4 Department of Agronomy, Purdue University, Lafayette, Indiana, United States of America; 5 Department of Statistics, Purdue University, Lafayette, Indiana, United States of America; 6 Cold Spring Harbor Laboratory, Cold Spring Harbor, New York, United States of America; 7 Ontario Institute for Cancer Research, Toronto, Ontario, Canada; University of Hong Kong, China

## Abstract

Recent data have revealed that epigenetic alterations, including DNA methylation and chromatin structure changes, are among the earliest molecular abnormalities to occur during tumorigenesis. The inherent thermodynamic stability of cytosine methylation and the apparent high specificity of the alterations for disease may accelerate the development of powerful molecular diagnostics for cancer. We report a genome-wide analysis of DNA methylation alterations in breast cancer. The approach efficiently identified a large collection of novel differentially DNA methylated loci (∼200), a subset of which was independently validated across a panel of over 230 clinical samples. The differential cytosine methylation events were independent of patient age, tumor stage, estrogen receptor status or family history of breast cancer. The power of the global approach for discovery is underscored by the identification of a single differentially methylated locus, associated with the *GHSR* gene, capable of distinguishing infiltrating ductal breast carcinoma from normal and benign breast tissues with a sensitivity and specificity of 90% and 96%, respectively. Notably, the frequency of these molecular abnormalities in breast tumors substantially exceeds the frequency of any other single genetic or epigenetic change reported to date. The discovery of over 50 novel DNA methylation-based biomarkers of breast cancer may provide new routes for development of DNA methylation-based diagnostics and prognostics, as well as reveal epigenetically regulated mechanism involved in breast tumorigenesis.

## Introduction

Breast cancer is the second leading cause of cancer-related deaths among women in the United States. Recent declines in breast cancer-associated mortality are partly attributed to the use of screening mammography, however, the benefit is significant only for women over 40 years of age [1], [2]. Approximately 33% of breast cancers detected by screening mammography represent overdiagnosis, leading to unnecessary treatment [3]. Furthermore, false positive results are estimated to occur in 50% of women screened annually for 10 years, 25% of whom will go on to have biopsies [4] and false negative results are a major concern, especially in younger women [5], [6]. Recently, MRI has proved to be a superior method to detect breast cancer in high risk patients; however the improvement in detection comes at the cost of an increased number of false positive cases [7]. Therefore, there is a critical need for improved molecular biomarkers that are capable of detecting early stage disease, indicating recurrence of disease, as well as predicting the progression of benign high-risk lesions and intraductal carcinoma *in situ* to invasive carcinoma.

Genetic mutations in *BRCA1* and *BRCA2* [8], [9], *BRIP1* [10], *CHEK2* [11], *ATM* [12] and *TP53* [13], [14] result in increased risk of breast cancer. However, these are estimated to account for only 5% to 10% of breast cancer cases [15], [16], [17] A recent large-scale sequencing analysis of over 13,000 genes in a small collection of breast tumors identified 122 genes with somatic mutation frequencies higher than the background frequency. However, each tumor harbored only a few mutations, and no single mutation or combination of mutations predominated across the tumor samples [18].

In addition to genetic alterations, epigenetic abnormalities such as changes in genomic DNA cytosine methylation patterns are associated with all cancer types. The spectrum of alterations includes both gain and loss of DNA methylation involving multi-copy elements as well as single-copy genes (reviewed in [19]). Many of the changes affect gene expression and genome stability through inappropriate regulation of local chromatin structure (reviewed in [20]). Furthermore, recent data suggest that epigenetic changes are involved in the earliest phases of tumorigenesis, and that they may predispose stem/progenitor cells to subsequent genetic and epigenetic changes involved in tumor promotion [21]. Given the observed frequency of DNA methylation changes in tumorigenesis and the inherent stability of the molecular abnormality, these events may provide ideal biomarkers for molecular diagnostics and early detection of cancer.

Several genes have previously been shown to be aberrantly methylated in breast cancer (reviewed in [22]). The majority of these have been identified through candidate gene approaches, and their observed frequency and disease specificity vary between independent studies. For example, *RASSF1A* is among the most commonly reported differentially methylated genes for numerous cancer types. Comparing two independent studies of *RASSF1A* methylation in breast cancer, the average frequency at which hypermethylation was detected in breast tumors is 56% [23], [24]. Approaches for genome-wide DNA methylation analysis hold promise to identify novel epigenetic targets with improved clinical sensitivity and specificity and, therefore, provide superior candidates for development of DNA methylation-based molecular diagnostics.

We have applied a microarray-based strategy for comprehensive DNA methylation profiling to discover differentially methylated loci in breast cancer. The approach is based upon the loose site specificity (purine-5mC) of the cytosine methylation dependent restriction enzyme McrBC and, therefore, is capable of determining the regional DNA methylation density associated with the plurality of molecules present. In the present study, the approach revealed numerous novel epigenetic biomarkers capable of distinguishing infiltrating ductal breast carcinomas from normal and benign breast tissues. A subset were extensively validated by screening a panel of over 230 clinical samples, revealing biomarkers that display clinical sensitivity and specificity up to 90% and 96%, respectively. Bisulphite sequencing analyses confirmed the DNA methylation changes and validated the qPCR-based assay adapted for high-throughput DNA methylation screening. In addition to identifying exceptionally promising biomarkers for improved disease detection, the functions of the associated genes suggest that the approach may also provide critical insights into molecular mechanisms of breast tumorigenesis.

## Results

### Genome-Wide Approach Identifies High-Frequency DNA Methylation Changes

**Figure 1:**
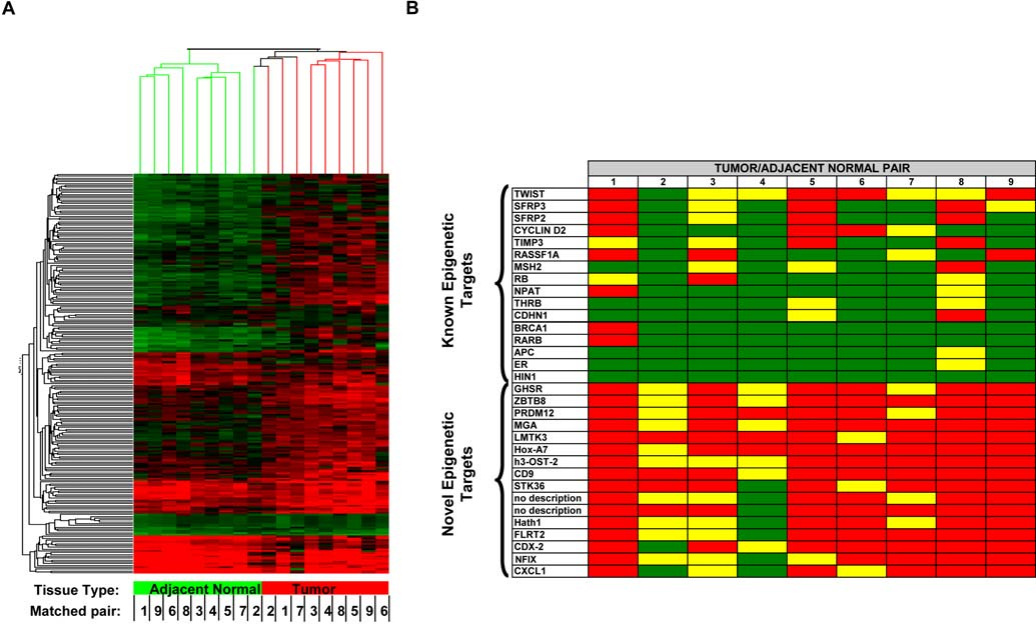
Comparison of previously identified and novel epigenetic targets in breast cancer. (A) Hierarchical clustering of differentially methylated loci identified by microarray analysis. DNA methylation measurements of 220 loci identified to be significantly differentially methylated by statistical analysis of global DNA methylation profiles are shown. The color scale of the heatmap represents densely methylated loci (red) to sparsely methylated loci (green). Unsupervised clustering (top dendrogram) distinguishes adjacent histology normal breast tissues (green branches) from breast cancer tissues (red branches). Individual matched tumor/adjacent histology normal tissues pairs are indicated by a number assigned to each individual (Matched pair). (B) Differential DNA methylation in individual tumor and adjacent histology normal tissue pairs. Differences between log_2_ ratios for individual tumor and adjacent histology normal pairs are shown for known and novel epigenetic targets. Because the experimental procedure compares total genome representations to those depleted for fragments containing DNA methylation, methylated sequences have a untreated:depleted ratio near or above 1.0 while unmethylated sequences have a ratio approaching zero due to mass normalization of target DNA [27]. Log_2_ differences ≥0.7 (red), 0.5 to 0.6 (yellow) and ≤0.5 (green) are shown. The annotated genes associated with the differential DNA methylation events are indicated at the left.

Genome-wide DNA methylation analysis at more than 21,000 loci was performed in nine infiltrating ductal breast carcinoma (IDC) and nine patient-matched adjacent histology normal samples. The IDC panel included six stage IIA and three stage IIB tumors from women ranging from 32 to 57 years of age (median age 47). Tumor and adjacent normal samples contained ≥65% and 0% neoplastic cellularity by H&E histology, respectively. Tumor samples included three IDCs positive for both estrogen and progesterone receptors and six IDCs negative for both receptors. Demographic information for these patients is provided in Table S1. DNA methylation profiles were generated using the previously described McrBC-based approach [25], [26], [27], 

Following statistical analyses, 220 loci were identified that provided optimal distinction between tumor and adjacent normal DNA samples. As expected, unsupervised hierarchical clustering of the data derived from these microarray features divided samples into two major clusters (Fig. 1A). All nine tumor samples were grouped into one major cluster, while eight of nine adjacent normal samples were grouped into the other cluster. The adjacent normal sample assigned to the tumor cluster was most closely related to its matched tumor sample (matched pair 2). The identified differentially methylated loci included both hyper- and hypomethylation events in tumor relative to adjacent normal samples. Raw and normalized array data for these loci are provided in Table S2.

A powerful novel discovery approach should recapitulate previous findings, as well as identify novel molecular abnormalities that offer advantages relative to what is known. The log_2_ ratios of DNA methylation measurements for 16 genes reported to be differentially methylated in breast cancer are depicted in Fig. 1B (“Known Epigenetic Targets”). By way of comparison, 16 novel differentially methylated loci discovered in this experiment are also shown (corresponding to the 16 loci described in Table 1; “Novel Epigenetic Targets”). Although the known epigenetic targets were occasionally differentially methylated, the novel epigenetic targets were differentially methylated in a larger number of tissue pairs. Importantly, for tissue pairs in which the loci reported differential methylation, the load of differentially methylated molecules reported was not significantly different for the known and novel targets (data not shown), indicating that there was no difference in the ability of the respective microarray features to report DNA methylation changes. For known epigenetic targets, differences between previously reported frequencies of DNA methylation changes and those predicted by this discovery experiment may be a consequence of the relatively small sample size or technical differences in the methods used to detect DNA methylation.

**Table 1:** Breast Cancer Biomarker Validation.

	BREAST TUMOR VS. NORMAL BREAST					BREAST TUMOR VS. NORMAL BLOOD		
Description	Sensitivity	Pos. (Total)	Specificity	Neg. (Total)	Threshold^b^	Specificity	Neg. (Total)	Threshold^b^
GHSR	90%	92 (102)	96%	99 (103)	0.64	100%	24 (24)	1.22
no description (chr7-8256880)^a^	89%	90 (101)	92%	96 (104)	0.555	100%	25 (25)	0.695
LMTK3	77%	78 (101)	87%	89 (102)	0.755	96%	23 (24)	1.06
MGA	70%	69 (99)	92%	86 (93)	1.11	95%	18 (19)	0.935
no description (chr1-203610783)^a^	69%	69 (100)	82%	84 (103)	0.615	87%	20 (23)	0.63
CD9	65%	66 (102)	97%	101 (104)	0.705	100%	25 (25)	0.535
hATH1	63%	64 (101)	97%	101 (104)	0.525	100%	25 (25)	0.57
STK36	63%	64 (101)	93%	96 (103)	0.5	71%	17 (24)	0.55
h3-OST-2	60%	61 (101)	98%	102 (104)	0.51	96%	23 (24)	0.51
FLRT2	58%	59 (102)	100%	104 (104)	0.515	100%	23 (23)	0.515
PRDM 12	56%	51 (91)	97%	96 (99)	0.545	95%	20 (21)	0.545
NFIX	53%	52 (98)	97%	94 (97)	0.61	96%	23 (24)	0.855
CDX-2	48%	49 (103)	97%	100 (103)	0.51	100%	24 (24)	0.51
CXCL1	42%	42 (101)	99%	98 (99)	0.545	100%	24 (24)	0.71
ZBTB 8	38%	39 (103)	97%	101 (104)	0.5	100%	25 (25)	0.72
Hox-A7	34%	35 (102)	97%	100 (103)	0.535	100%	22 (22)	0.535

a Loci not within the vicinity of a known annotated gene (no description) are described by chromosome number and nucleotide position of the microarray feature (Ensembl 36).

b Thresholds indicate the optimal average dCt value for distinction between tumor and non-tumor tissues.

**Figure 2:**
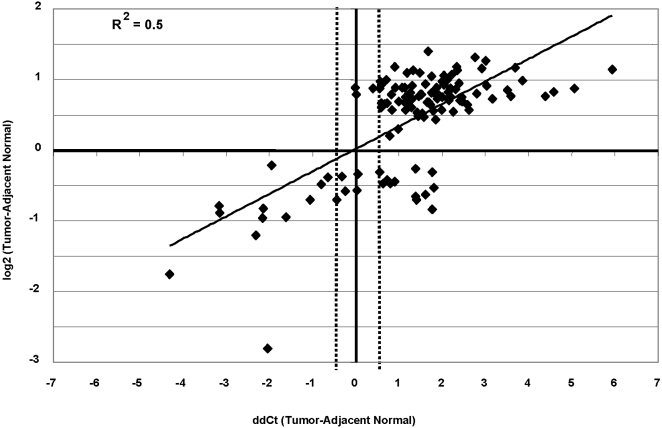
Correlation between differential DNA methylation measured by microarray and independent qPCR analyses. Log_2_ (tumor-adjacent normal) microarray measurements of differential DNA methylation (y-axis) are plotted against qPCR (ddCt tumor-adjacent normal) measurements (x-axis). Primer pairs designed to amplify 116 of the 220 regions predicted to be significantly differentially methylated. Delta-delta Ct values (delta Ct _tumor_–delta Ct _adjacent normal_) and differential log_2_ microarray values (Log_2 tumor_-Log_2 adjacent normal_) were compared for breast tumor/adjacent normal tissue pairs. Data for one representative tumor/adjacent normal pair are shown. Data points in the upper right and lower left quadrants represent hypermethylation and hypomethylation measurements that are concordant between the two independent methods, respectively. qPCR measurements within the 0.5 cycle range of variance of the qPCR platform (hatched lines) were considered discordant.

Accuracy of the microarray-based DNA methylation measurements was assessed by a quantitative PCR (qPCR) [27] as described in the Materials and Methods. The presence of purine-5mC sites within an amplified region results in digestion by McrBC and a higher cycle number at which the McrBC-treated sample crosses threshold. Therefore, higher delta Ct measurements correlate with a larger proportion of the molecules containing DNA methylation between the priming sites (Fig. 2). In total, 96 of 116 (83%) measurements were concordant between the two methods (12 hypomethylation and 84 hypermethylation events). Nine qPCR measurements fell within the 0.5 cycle region of variability of the real-time PCR platform itself, and these measurements were considered discordant. Seventeen measurements reported an increase in DNA methylation by the qPCR method that was not detected by the microarray method (approximately 15% false negative rate in the microarray experiment). Three hypermethylation microarray predictions fell within the 0.5 cycle qPCR range that was considered discordant (approximately 2% false positive rate). An overall accuracy of 83% and a higher false negative than false positive rate are consistent with results obtained in numerous independent microarray analyses ([27] and data not shown). A precisely linear relationship between the microarray and qPCR measurements is not necessarily expected because the microarray features are capable of measuring methylation of a larger local region (1 to 4 Kb) than the qPCR amplicons were designed to interrogate (400–600 bp).

### Validation of Differential DNA Methylation Events in Large Panels of Clinical Tissue Samples

**Figure 3:**
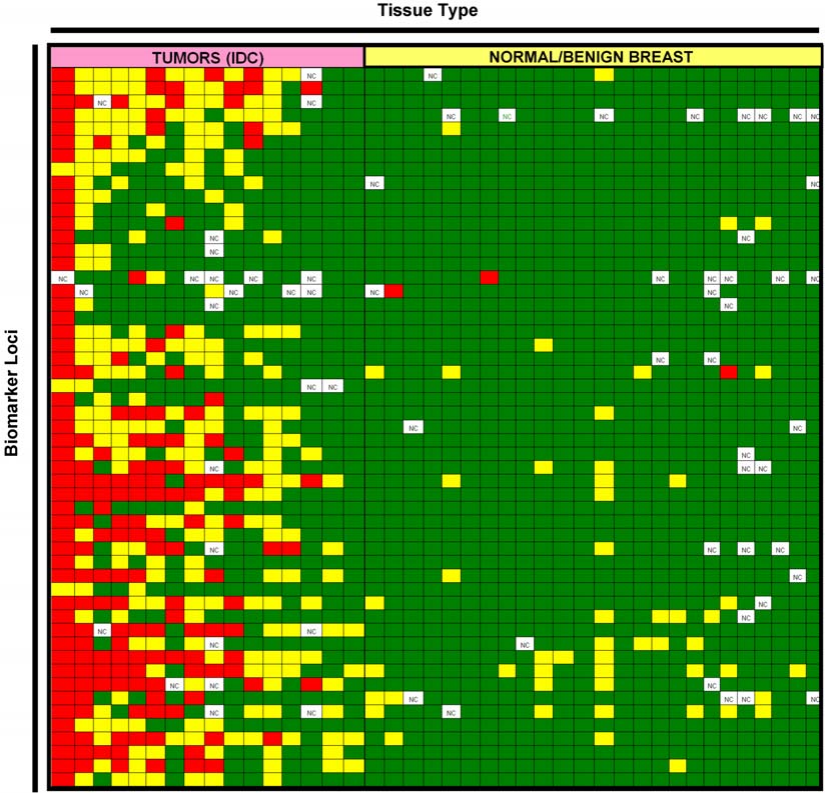
Differential DNA methylation of identified loci within an initial validation panel of clinical samples. qPCR measurements of DNA methylation density were obtained for 53 loci across 16 IDC tumor samples and 25 normal or benign breast samples. An average delta Ct (Ct _McrBC_–Ct _Mock_) less than 1.0 was scored as sparsely methylated (green cells). A delta Ct of 1.0 indicates that approximately half of the DNA in the reaction was cleaved by McrBC within the amplified region and therefore contained a measurable density of DNA methylation. An average delta Ct greater than or equal to 1.0, but less than 2.0 was scored as intermediately methylated (yellow cells). Finally, an average delta Ct≥2.0 (≥75% of DNA molecules were cleaved by McrBC) was scored as densely methylated (red cells).

As an initial validation, qPCR assays for 53 loci hypermethylated in at least 70% of tissue pairs were conducted across a panel of 16 independent IDCs (Stage II) and 25 normal or benign breast tissues. We focused on hypermethylation events because hypomethylation events were considerably less frequent than hypermethylation events (*i.e.* <40% sensitivity). This observation has been made previously by Bestor and colleagues (A. O'Donnell, R. Rollins, and T.H. Bestor (personal communication)). As shown in Fig. 3, differential DNA methylation between tumor and non-tumor breast samples was confirmed in an independent tissue panel. The differentially methylated regions displayed a range of clinical sensitivity (*i.e.* the percentage of tumors displaying intermediate to dense DNA methylation) and clinical specificity (*i.e.* the percentage of normals displaying sparse DNA methylation). Among loci displaying 100% specificity relative to normal breast tissue, sensitivities ranged from 6% (1 of 16 tumors were methylated; *IGF-II mRNA binding protein 3*) to 81% (13 of 16 tumors were methylated; *GHSR*) (Table S3). Because these differentially methylated loci may be useful for disease detection in peripheral fluids such as plasma or serum, the methylation status of each locus was analyzed in a panel of 19 blood samples from cancer-free women. Although the majority of the 53 loci demonstrated greater than 80% specificity relative to normal peripheral blood, 21 loci (40%) were methylated in at least half of the normal blood samples (Table S3). Therefore, these results indicate that a subset of loci that become hypermethylated in breast cancer take on a DNA methylation state that is similar to the normal methylation state in circulating blood cells. Similar results were obtained in a recent DNA methylation analysis of lung tumors and peripheral blood [28]. Although the biological mechanisms and consequences of the DNA methylation similarities between tumor and normal peripheral blood cells are yet to be determined, these findings are important in terms of the applicability of differentially methylated loci for use as potential biomarkers for early detection of cancer using peripheral fluids such as serum or plasma

The 16 differentially methylated regions that displayed greater than 95% specificity relative to normal and benign breast tissue and normal blood were analyzed in a larger validation panel, including a total of 103 IDC samples (8 Stage I, 65 Stage IIA, 28 Stage IIB, 2 Stage III), 104 normal or benign breast samples and 25 peripheral blood samples from cancer-free women (Table 1). Demographic information for the patients included in this panel is provided in Table S4. Sensitivity and specificity calculations across the expanded validation panel were consistent with those obtained from the initial panel. Of the 103 IDC samples, 96 scored as methylated for at least one of the 16 differentially methylated loci (93%), and 94 (91%) scored as methylated for more than one locus. The seven samples that scored as sparsely methylated for all 16 loci included 4 Stage IIA tumors and 3 Stage IIB samples. Therefore, the absence of hypermethylation at these loci was not exclusively associated with the earliest tumor stage. Furthermore, patient age was not associated with the lack of differential methylation (p = 0.804, t test). These tumors may represent a minor subclass that do not undergo extensive epigenetic rearrangements or that undergo a different epigenetic alteration program than the majority of tumors. Global DNA methylation profiling of these tumors directly may identify alternative tumor-specific epigenetic abnormalities common to this small group.

**Figure 4:**
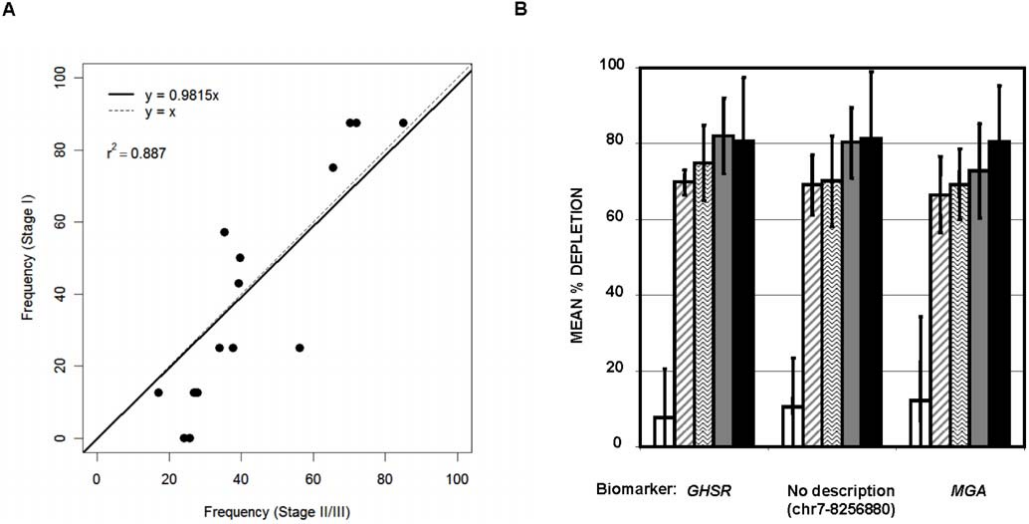
Differential DNA methylation relative to tumor stage. (A) Comparison of the frequency of differential DNA methylation of 16 loci in stage I breast tumors relative to stage II–III breast tumors. (B) DNA methylation density of 3 selected loci relative to tumor stage. The percent depletion by McrBC for each sample in which a given locus scored as methylated was calculated [1-(1/2̂delta Ct (McrBC digested – Mock treated)) * 100] to provide a measure of the load of methylated molecules within the sample. The % depletion is plotted (from left to right) for normal and benign samples, stage I tumors, stage IIA tumors, stage IIB tumors and stage III tumors.

Fig. 4A shows a plot of the frequency of hypermethylation of the 16 loci in the 8 Stage I tumors (*i.e.* the percentage of Stage I tumors scoring as intermediately to densely methylated) versus the Stage II and III tumors. The directly proportional relationship between the two sensitivity calculations (R^2^ = 0.887; slope = 0.9815) indicates that the frequency of hypermethylation of these loci is similar regardless of tumor stage. Therefore, for the majority of loci, the differential methylation events are just as likely to be present in a Stage I tumor as they are in later stage tumors. The proportion of methylated molecules in tumors at each stage was then analyzed for three selected loci (Fig. 4B). While there was a trend for increased methylation density at these loci with increasing tumor stage, methylation density of Stage I tumors was not significantly different than Stage II–III tumors, yet dramatically different than normal samples. Therefore, differential methylation of these loci is independent of tumor stage in regards to both frequency and density of hypermethylation.

### Novel Differential DNA Methylation Events Display Exceptionally High Sensitivity and Specificity for Breast Tumors

**Figure 5:**
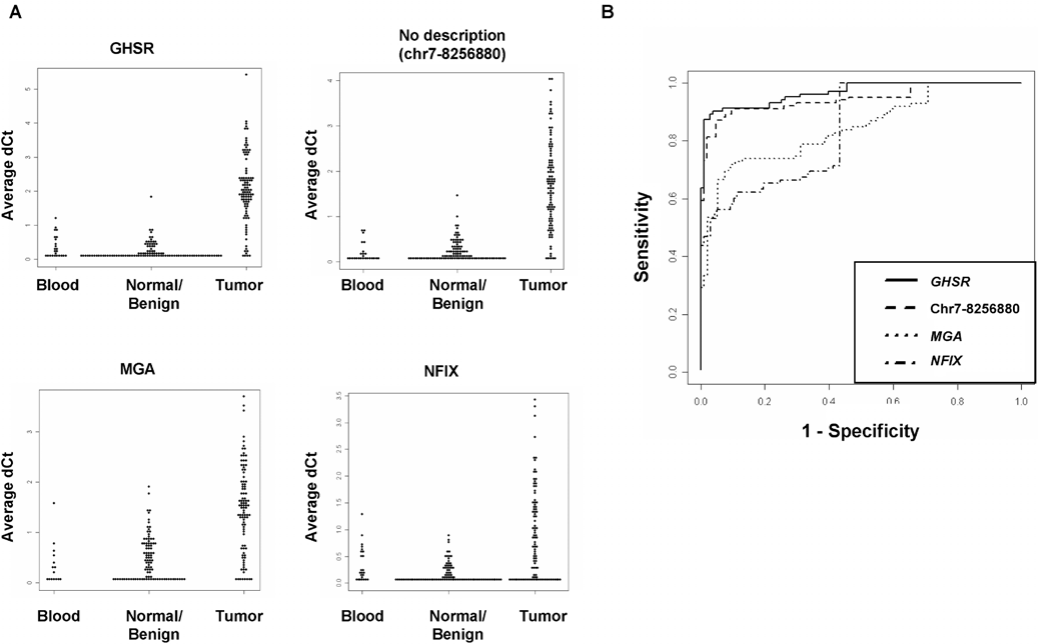
Differential DNA methylation of selected loci. (A) DNA methylation measurements for selected loci in normal blood, normal or benign breast tissue and breast tumors. Each data point represents the averaged delta Ct value for an independent clinical sample. (B) ROC curve analyses of the four loci shown in (A). Sensitivity (percentage of tumor samples scoring above a methylation threshold) and specificity (percentage of non-tumor samples scoring below that same threshold) were calculated for all observed delta Ct values. The minimum allowed threshold was set at 0.5 so that calculations could not be based on thresholds within the variability range of the qPCR platform.

Receiver-operator characteristic (ROC) analysis was performed for each of the 16 loci to determine optimal thresholds for calculation of sensitivity and specificity of the differential DNA methylation event. Examples of the primary qPCR data for four selected loci are shown in Fig. 5A. The frequency at which tumor tissues were scored as differentially methylated at these loci was not significantly associated with patient, age, estrogen receptor status or family history of breast cancer (data not shown). BRCA1 or BRCA2 mutation status was unknown for these patients. ROC curves for the corresponding four datasets are shown in Fig. 5B. Optimal thresholds were identified as the maximum sum of sensitivity and specificity calculated at each observed delta Ct value. Results are summarized in Table 1. Sensitivity and specificity calculations based on optimal thresholds are similar to those calculated using a standard delta Ct threshold of 1.0 (compare Table 1 and Table S4). As hypothesized, the direct global profiling of DNA methylation identified numerous novel DNA methylation-based biomarkers that display substantially improved sensitivity and specificity relative to the vast majority of previously identified differentially methylated genes in breast cancer. In fact, a single differentially methylated biomarker, located in the upstream region of *GHSR*, was capable of distinguishing IDC from normal and benign breast tissue with sensitivity of 90% and specificity of 96%.

Quantitative PCR (like other technical platforms) involves an inherent level of technical variability. This variability could potentially have an impact on calculated sensitivity and specificity when ROC-based thresholds are close to the 0.5 cycle variability range of the qPCR platform. We addressed this issue first by performing technical replicates of all measurements, and excluding measurements with standard deviations ≥1.0. To further investigate the impact of this variability, we repeated the *GHSR* qPCR measurements for the 16 samples near the 0.64 dCt threshold (0.5–1.5). Three replicates were preformed using a different lot of McrBC. All 11 tumor samples again scored above the dCt threshold in all three replicates (Fig. S1). Three of five normal samples that originally scored just above the dCt threshold scored below the threshold in all three replicates. Therefore, repeating measurements near the ROC established thresholds had no impact on the calculated sensitivity, but had a relatively minor impact on specificity (increased from 96% to 99%).

Other biomarkers displayed similar specificity, with decreasing sensitivity. Discriminant analysis was performed (including all 16 biomarkers screened against 103 IDC samples and 104 normal breast tissues) to identify potential biomarker panels with greater sensitivity and specificity than methylation of *GHSR* alone. Models including one to four biomarkers were constructed and tested by determining the error rate of classifying tumor and normal samples in a leave-one-out cross-validation paradigm. As expected, methylation of *GHSR* was the optimal single biomarker, resulting in an overall average error rate of 8.3% (16 of 103 tumors were misclassified as normal, and 1 of 104 normals was misclassified as tumor). No two- or three-biomarker panel reduced this error rate. Six four-biomarker panels resulted in less than 1% reduction in error rate. Therefore, biomarker combinations did not result in a biologically significant increase in sensitivity and specificity relative to that of differential DNA methylation of the *GHSR* locus alone. Future experiments aimed at direct identification of epigenetic abnormalities associated with the minor class of tumors that were not hypermethylated at *GHSR* (∼10% of the total tumors analyzed) may identify low frequency differential methylation events that, when combined with *GHSR* hypermethylation, lead to sensitivity approaching 100%.

### Differential DNA Methylation Events are Confirmed by an Independent Approach

**Figure 6:**
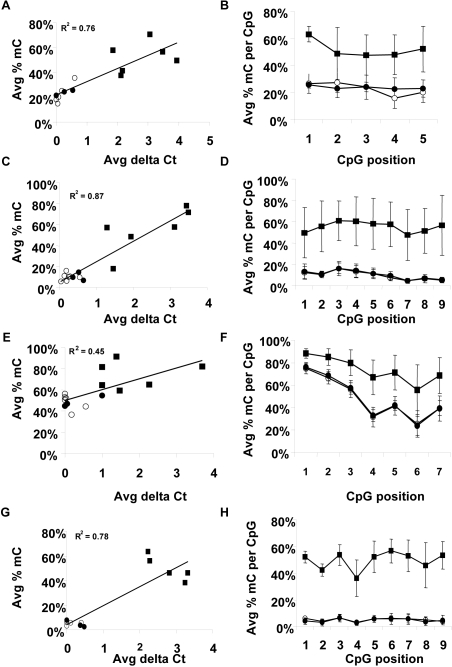
Bisulphite sequencing analysis of DNA methylation. Analyzed loci included *GHSR* (A, B), the uncharacterized locus corresponding to chr7-8256880 (C, D), *MGA* (E, F) and *NFIX* (G, H). Bisulphite sequencing was performed by 454 Life Sciences technology as described in Materials and Methods. The average number of molecules sequenced for each locus within each sample was 587. The calculated DNA methylation density (number of methylated CpGs divided by the total number of CpGs sequenced) for each sample is plotted versus the qPCR DNA methylation measurement for the same sample (A, C, E, G). Analyzed samples included normal breast tissues (open circles), tumor-adjacent histology normal breast tissues (filled circles) and breast tumors (filled squares). The number of samples analyzed and the qPCR-based methylation score are as follows: *GHSR,* 6 tumor (5 densely and 1 intermediately methylated), 3 adjacent normal (sparsely methylated) and 5 normal (sparsely methylated); chr7-8256880, 6 tumor (3 densely and 3 intermediately methylated), 3 adjacent normal (sparsely methylated) and 7 normal (sparsely methylated); *MGA,* 6 tumor (2 densely and 4 intermediately methylated), 3 adjacent normal (2 sparsely and 1 intermediately methylated) and 5 normal (sparsely methylated); *NFIX*, 5 tumor (densely methylated), 3 adjacent normal (sparsely methylated) and 5 normal (sparsely methylated). In addition, the percent methylation occupancy at each analyzed CpG dinucleotide is shown (B, D, F, H).

To provide an in-depth analysis of DNA methylation states relative to the qPCR-based measurements of methylated DNA, we selected four loci (*GHSR, MGA, NFIX* and the uncharacterized region corresponding to chr7-8256880 (UCSC hg.18(NCBI36)) for extensive bisulphite sequencing analysis (Fig. 6). Analyzed sequences overlapped those amplified in the qPCR assay. For analysis of each locus, we selected five to six tumor samples that scored as intermediately to densely methylated and five to seven normal breast samples that scored as sparsely methylated. In addition, we selected three adjacent histology normal tissue samples. Massively parallel bisulphite sequencing was performed as described in the Materials and Methods. The average number of molecules analyzed for each locus in each sample was 587. To provide a general measurement of local DNA methylation density at each locus, the total number of CpG sites sequenced as C (methylated) was divided by the total of number of CpG sites sequenced for each individual sample. This percent methylated CpG value was then plotted against the qPCR methylation measurement for the same tissue sample (Fig. 5A, C, E, G). Methylation load values obtained by bisulphite sequencing and by qPCR displayed a strong correlation for *GHSR, NFIX* and the uncharacterized region corresponding to chr7-8256880 (R^2^ = 0.76, 0.87 and 0.78, respectively). While tumor samples displayed higher DNA methylation load at *MGA* than normal breast and adjacent histology normal breast samples, the non-tumor tissues displayed higher baseline DNA methylation densities than at the other loci (Fig. 5E). Next, the average occurrence of DNA methylation per CpG site in each tissue type was calculated (Fig. 5B, D, F, H). In general, tumor samples displayed higher variability in methylation per CpG site than non-tumor samples (indicated by higher standard deviations for the average percent methylated CpGs). At each locus, the DNA methylation pattern was significantly hypermethylated relative to non-tumor samples. Furthermore, analysis of DNA methylation per CpG site provided an explanation for the higher non-tumor baseline DNA methylation densities detected at the *MGA* locus (Fig. 5F). In non-tumor samples, methylation densities at the first three CpG dinucleotides of the analyzed region were greater than 50%, while methylation of the following four CpG dinucleotides fell to lower densities more consistent with the baseline levels at the other analyzed loci. Interestingly, tumor samples displayed the same general methylation density pattern, but with significantly higher methylation density per CpG across the entire analyzed region. Together, these results confirm the hypermethylated state of these loci in breast cancer and provide an extensive validation of the accuracy of the qPCR-based method used to screen for DNA methylation changes.

### *GHSR* Hypermethylation Correlates with Decreased Gene Expression

**Figure 7:**
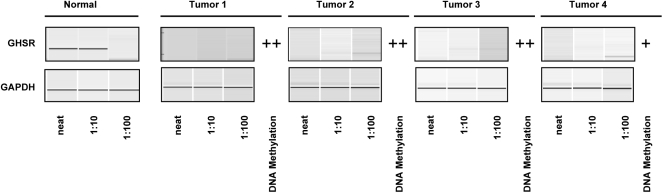
Correlation between DNA hypermethylation and gene expression. Transcription of *GHSR1a* was analyzed by RT-PCR. RT-PCR was performed using gene-specific primer pairs designed to flank intronic sequence so that the contribution of contaminating genomic DNA could be excluded. Analysis of *GAPDH* expression was performed as an internal control. Serial dilutions of first-strand cDNA preparations from tumor samples and a normal breast tissue sample were used as templates for PCR. The DNA methylation measurement (qPCR) for each locus in each tumor sample is indicated (- sparse, + intermediate and ++ dense methylation).

To address the association between hypermethylation and transcription repression, we performed RT-PCR analyses of the *GHSR* gene (Fig. 7). Four breast IDC samples (>90% neoplastic cellularity) were analyzed for both DNA methylation and transcription. Expression of *GHSR1a* (see Discussion) was undetectable in all four tumor samples, while expression was detected at 1∶10 dilution of the normal breast cDNA. Consistent with the high sensitivity of hypermethylation at the *GHSR* locus, all four tumor samples demonstrated intermediate to dense DNA methylation at this locus.

## Discussion

We have combined the use of high-content DNA microarrays designed specifically for analysis of DNA methylation patterns with a sensitive DNA methylation-dependent enzymatic approach to generate genome-wide DNA methylation density profiles in breast tumor and unaffected breast tissue. This approach proved to be both efficient and effective, in that global profiling of a small number of tissue samples identified several novel epigenetic targets that were extensively validated within a panel of over 230 clinical samples.

Strikingly, the approach identified a single locus within the promoter region of the *GHSR* gene that is hypermethylated in 90% (92 of 102) of infiltrating ductal breast carcinomas, independently of patient age or tumor stage. Conversely, 4 of 103 cancer-free breast tissues reported a DNA methylation density measurement slightly above the ROC curve-established threshold (96% specificity). To our knowledge, this locus represents the highest combined sensitivity and specificity for a DNA-based (genetic or epigenetic) biomarker of breast cancer reported to date.

Clinical applications of these DNA methylation based biomarkers range from early, possibly non-invasive cancer detection to more accurate molecular classification of confirmed breast cancers. For example, we focused on novel biomarker loci displaying high clinical specificity for disease, yet the level of sensitivity of the differential DNA methylation events vary among the loci. Those with exceptionally high sensitivity are candidates for future development of screening diagnostics for the early detection of cancer and for the prediction of progression of high risk lesions such as ductal or lobular carcinoma *in situ* to malignancy. Notably, we have confirmed the ability to detect tumor-associated hypermethylation of several loci in fine needle aspirate specimens collected from breast cancer patients. The frequency of detection of hypermethylation of these loci is similar regardless of whether the tested DNA is derived from primary tumors or from fine needle aspirates (Fig. S2). The detection of circulating tumor-associated DNA methylation-based biomarkers in serum has also been explored for early, non-invasive screening applications. However, the molecular complexity of DNA methylation patterns in sera from cancer-free individuals indicates that a comprehensive understanding of precise methylation configurations is essential for the future development of such diagnostics [29]. On the other hand, biomarker loci that display high specificity yet lower sensitivity are candidates for potential applications aimed at sub-classifying tumors by disease prognosis or responsiveness to certain therapies. Given the exceptional performance of these novel biomarkers within this discovery and validation study, analyses of clinical samples collected to specifically address these applications are clearly warranted.

In addition to revealing novel and powerful epigenetic biomarkers of breast cancer, our results provide insights into potential epigenetic mechanisms of breast tumorigenesis. As described above, hypermethylation of the promoter region of *GHSR* was detected in primary breast tumors at an exceptionally high frequency. Furthermore, reduced *GHSR 1a* mRNA expression was associated with hypermethylation. *GHSR* encodes a seven transmembrane-spanning G protein-coupled receptor (GHSR 1a) for the circulating peptide hormone, ghrelin. A second transcript encodes a truncated form of the receptor (GHSR 1b), presumably via alternative splicing and polyadenylation within the single intron [30]. However, transcripts initiating from upstream transcriptional start sites have also been reported [31]. Recent evidence suggests that alterations of the ghrelin/GHSR axis may play an important autocrine/paracrine role in hormone-dependent cancers (reviewed in [32]), however potential mechanisms appear to be complex. For example, a recent immunohistochemistry (IHC) study indicated that the GHSR 1b isoform is undetectable in normal breast tissue, but dramatically up-regulated in all breast tumors analyzed [33]. The GHSR 1a isoform was detected by IHC in the cytoplasm of glandular epithelial cells of breast tumor tissue. However, RT-PCR analyses of breast tumor cell lines demonstrated dramatic reduction of *GHSR 1a* mRNA expression in 3 of 4 cell lines tested (consistent with the frequency of *GHSR* hypermethylation reported here). Loss of GHSR 1a expression and overexpression of GHSR 1b has also been reported in adrenocortical carcinomas [34]. Whether the high-frequency hypermethylation event detected at the *GHSR* locus is involved in the switch between GHSR 1a and GHSR 1b isoform expression, potentially by directing a switch to alternative upstream transcription start sites, is a focus of future research. Furthermore, since GHSR 1b is reported to be an inactive isoform of the receptor [35], loss of expression of GHSR 1a and overexpression of GHSR 1b may be functionally equivalent mechanisms of altering the ghrelin/GHSR axis in breast cancer.

Another high-frequency hypermethylation event is associated with the *MGA* gene, encoding a transcriptional repressor that intersects with the Myc pathway. Myc gene amplification occurs in approximately 15–20% of breast cancer patients [36] and has been reported to be an independent predictor of survival in patients treated with tamoxifen [37]. Overexpression of c-*myc* occurs in up to 50% of tumors [38], [39], [40]. When heterodimerized with Max, Myc regulates the expression of numerous target genes involved in aspects of tumorigenesis including cell cycle regulation, cell growth, cell adhesion, immortalization and genomic stability (reviewed in [41]). MGA (Max Gene Associated) also forms hetero-dimers with Max to form a transcriptional repressor co-complex that antagonizes the activity of Myc. MGA-Max complexes have been identified as part of the E2F6 repression complex that occupies and represses E2F- and Myc-responsive promoters at G_0_ [42]. Therefore, epigenetic repression of MGA expression may contribute to breast tumorigenesis by shifting the balance between activating and repressing signals upstream of Myc function. Importantly, ectopic expression of MGA can block Myc-dependent cellular transformation in cell culture assays [43], implying that it may itself provide a tumor suppressor function *in vivo*. The frequency of MGA hypermethylation in breast tumors suggests that it may serve to accentuate the activity of Myc, even in the absence of Myc amplification or overexpression. Future studies of the association between MGA hypermethyaltion, transcriptional repression and Myc amplification/overexpression may reveal directly cooperative mechanisms involving oncogene activation and epigenetic inactivation of negative regulators of oncogene activity.

Finally, our approach identified previously uncharacterized loci subject to aberrant DNA methylation in breast cancer. For example, the uncharacterized locus corresponding to chr7-8256680 was hypermethylated at a similar frequency to *GHSR* (90 of 101 tumors; 89%). This region is located on chromosome 7, from approximate nucleotide 8449665 to 8450724 (hg18 (Ensembl 43, NCBI 36)), and includes an annotated CpG island. The region is devoid of confirmed genes within approximately 0.2 Mb upstream or downstream of this sequence, however the sequence falls within the 3′ regions of two juxtaposed predicted transcripts (Fig. S3). The exceptionally high sensitivity and specificity of this differentially methylated locus for breast cancer suggests that it imparts some functional consequence. Therefore, our approach for DNA methylation profiling may ultimately lead to the discovery of previously uncharacterized genomic elements that are important in tumorigenesis.

## Materials and Methods

### Tissues and Nucleic Acid Preparations

Breast tissue samples were obtained from Genomics Collaborative Institute (Essex IRB, Protocol Number 99-501.04) or provided through North Glasgow NHS Trust. Whole blood samples were obtained from Research Blood Components. All samples were collected with appropriate institute ethics approval and written consent was provided by each patient. Demographic and source information for patients included in this study is provided in Tables S1 and S4. Neoplastic cellularity of all breast samples was confirmed by H&E histology. Genomic DNA from both breast tissues and whole blood samples was extracted with the MasterPure DNA extraction kit (EpiCentre) by the manufacturer's protocol. For gene expression studies, 4 IDC samples were homogenized in PBS and split into two portions. Genomic DNA was extracted from one portion with the MasterPure DNA extraction kit. Total RNA was extracted from the second portion with TRIzol reagent (Invitrogen) by the manufacturer's protocol.

### DNA Methylation-Dependent DNA Fractionation, Microarray Hybridizations and Data Analysis

DNA samples were fractionated based on DNA methylation density as previously described [27]. Briefly, 60 µg DNA was mechanically sheared into a uniform 1 to 4 Kb molecular weight distribution (GeneMachines HydroShear) and split into 4 equal portions. Two portions (treated technical replicates) were digested with McrBC (NEB) in 150 µL total volume including 1× NEB2 buffer, 0.1 mg/mL bovine serum albumin, 2 mM GTP and 100 units McrBC. The remaining two portions were mock-treated under identical conditions except that 10 µL sterile 50% glycerol was added instead of McrBC. Following overnight digestion, reactions were treated with 5 µL proteinase K (50 mg/mL) for 1 hour at 50°C, and precipitated with EtOH under standard conditions. Samples were resolved on a 1% low melting point SeaPlaque GTG Agarose gel (Cambridge Bio Sciences, Rockland, ME). DNA within the modal size range of the untreated fraction (1–4 Kb) was excised and extracted from gel slices with Gel Extraction spin columns (Qiagen).

The OGHAv1.0 microarray has been previously described [27]. Microarray hybridization experiments were performed by NimbleGen Systems using a duplicated dye-swap design. The microarray data were analyzed with the objective of identifying differentially methylated regions between tumor and adjacent normal genomes. Convergence of independently derived gene-lists was utilized as a metric for target nomination. First, all adjacent normal and tumor data were separately normalized using a modified method of Yang et al [44]. The normalized data sets were analyzed using ANOVA and previously described methods [27] to identify differentially methylated regions between phenotypes. The second method did not employ normalization, rather each individual's adjacent normal sample was compared to the matching paired tumor tissue, providing a genetic control for individual-to-individual variation in DNA methylation. In this analysis, loci were nominated for each tissue pair, and those that were consistently selected across the nine matched pairs were identified. Analyses employed both per-gene and common variance and utilized the Holm and False Discovery Rate methods to control for multiple testing errors [45], [46]. Only microarray features that were significant in both the per-gene and common variance analyses were considered. Finally, a locus list was nominated based on the overlap between the previous two analyses. Detailed descriptions of statistical analysis methods, including normalization to control features representing loci lacking McrBC half sites, are available [27].

### Quantitative PCR Analysis of DNA Methylation

PCR primers were designed to amplify approximately 400–600 bp amplicons within a 1 Kb sequence window spanning the sequence represented by the associated microarray feature. Primer selection was guided by uniqueness of the oligonucleotide sequence across the human genome, as well as the CpG distribution within the 1 Kb sequence window. All primer pairs were confirmed to amplify a single product of appropriate size. Oligonucleotide sequences are provided in Table S5. DNA methylation was monitored by qPCR analysis of mock-treated and McrBC-digested portions of each sample, as previously described [27]. Breast and whole blood samples were treated identically. Genomic DNA samples (4 µg) were digested with McrBC (NEB) in 200 µL total volume including 1× NEB2 buffer, 0.1 mg/mL bovine serum albumin, 2 mM GTP and 32 units McrBC overnight at 37°C. Mock treatment was performed under identical conditions with the exception that sterile 50% glycerol was substituted for McrBC. All samples were incubated at 65°C for 20 min. to inactivate the McrBC. To avoid variation in DNA recovery, no further purification was performed prior to qPCR. 20 ng of each treatment was amplified in 10 µL volume including 1× SYBR Green Master Mix (Roche) and 625 nM each primer. All treatment pair reactions were performed in duplicate. qPCR was performed on the Roche LC480 system under the following conditions: Preamplification, 95°C for 5 min.; Amplification, 45 cycles of 95°C for 1 min., 66°C for 30 sec., 72°C for 1 min., 80°C for 2 sec. followed by a single acquisition; Metlting curve, 95°C for 5 min., 65°C for 1 min., ramped to 99°C at 2.5° per sec. with continuous acquisition. Digestion by McrBC was quality controlled by qPCR analysis of the promoter of the *TH2B* gene, which is densely methylated in all tissues except the testes [47]. All samples displayed a *TH2B* delta Ct≥4.0 (>90% depletion of the amplified region in the McrBC-digested portion relative to the mock-treated portion). The percent *TH2B* depletion for tumor, normal or benign breast tissue and blood samples did not differ significantly (p>0.5 for all pair wise combinations). For each McrBC-digested/mock-treated reaction pair, a delta Ct (Ct _McrBC_–Ct _Mock_) was calculated. Duplicate delta Ct values were averaged, and the standard deviations between delta Ct values were determined. All reported average delta Ct values had standard deviations less than 1 cycle.

### Bisulphite Sequencing Analyses

Bisulphite sequencing primers were designed to amplify DNA corresponding to the amplicons used for quantitative PCR analyses. Primer pairs (Table S5) flanked, but did not include, CpG dinucleotides [48]. For each analyzed DNA sample, 1–2 µg was bisulphite converted using EZ DNA Methylation Kits (Zymo Research) following the manufacturer's protocol. In-depth bisulphite sequencing was performed using the 454 Life Sciences platform. One oligonucleotide of each primer pair included a 5′patient-specific four-to-five base sequence tag [29]. Each tissue sample was amplified with a primer pair including a unique sequence tag. Amplicons were gel purified and quantified, then combined in equal molar concentrations. DNA sequencing was performed by the Washington University Genome Sequencing Center. To control for bisulphite conversion efficiency, incompletely converted molecules were identified and eliminated using the MethylMapper BisY control [49]. This excluded approximately 2% of the eligible reads. The next level of quality control assessed each read by ensuring that it exhibited a single-hit with a long high-scoring BLAST pair. Greater than 80% of the data passed both quality control metrics. Each amplicon and each patient were adequately represented in the final data collection, confirming that no single patient or amplicon dominated the analysis. MethylMapper BisT analysis [49] was used to generate DNA methylation data by CpG position and by molecule.

### *GHSR* Gene Expression Analyses

For IDC samples, 5 µg total RNA was used as template for cDNA synthesis by the Superscript III First Strand Synthesis System (Invitrogen) using the manufacturer's protocol. Prior to cDNA synthesis, RNA samples were treated with recombinant DNase I (Ambion) by the manufacturer's protocol. Normal breast cDNA was obtained from Invitrogen and was prepared using the same protocol. cDNA concentrations were normalized between samples and serial dilutions were used as template for PCR amplification. PCR primers (Table S5) were designed to flank intronic sequence so that amplification from contaminating genomic DNA could be excluded. Each reaction was performed in 10 µL total volume including 1× SYBR Green I Master mix (Roche) and 625 nM of each primer. PCR conditions for *GHSR* were as described [33]. Cycling conditions for *GAPDH* were 1 cycle of 95°C for 5 min., 30 cycles of 95°C for 30 sec., 65°C for 15 sec., 72°C for 15 sec., and 1 cycle of 72°C for 10 min. Amplification products (1 µL) were visualized and quantified using an Agilent Bioanalyzer 3100 and DNA 1000 LabChips (Agilent). All reactions were performed at least three times using two independently synthesized cDNA preparations.

## Supporting Information

Figure S1 Repeated GHSR qPCR analyses for samples near the 0.64 threshold for sensitivity and specificity calculations. 16 samples were analyzed three times (REP1, REP2, REP3). Digestions were performed using a different lot of McrBC enzyme than that used for the experiments summarized in Table 1 (ORIGINAL). Each value is an averaged dCt between two qPCR technical replicates. Samples scoring above the threshold are indicated in red, and those scoring below the threshold are indicated in green.
(0.01 MB PDF)

Figure S2 Detection of tumor-specific DNA hypermethylation in fine needle aspirate specimens. Eight biomarker loci were screened in seven FNA samples obtained from confirmed breast cancer cases. For each locus, the percentage of FNA samples that reported hypermethylation was plotted against the percentage of independent tumor samples that reported hypermethylation. If the biomarkers are detecting breast cancer at the same frequency as in tissue samples the expectation is that the two results should be directly proportional (i.e. exhibit a sensitivity slope of 1.0). This theoretical maxim is indicated by the dashed y = x line. The actual slope (solid line) and its R2 are indicated. The theoretical and experimental results are not significantly different (n = 8 data points).
(0.01 MB PDF)

Figure S3 Ensembl contig view of the uncharacterized locus corresponding to chr7-8256680(NCBI35). The position of the microarray feature that reported differential DNA methylation and Ensembl annotated CpG islands are indicated by arrows (NCBI36(hg18)).
(0.16 MB PDF)

Table S1 Patient demographics for samples used in microarray analyses
(0.04 MB DOC)

Table S2 Microarray data.
(1.06 MB XLS)

Table S3 Sensitivity/Specificity of Breast Cancer Biomarkers in Initial Validation Panel.
(0.03 MB XLS)

Table S4 Patient demographics for samples used in validation analyses.
(0.08 MB DOC)

Table S5 Oligo sequences
(0.03 MB XLS)

## References

[1] Nystrom L, Andersson I, Bjurstam N, Frisell J, Nordenskjold B (2002). Long-term effects of mammography screening: updated overview of the Swedish randomised trials.. Lancet.

[2] Miller AB, To T, Baines CJ, Wall C (2002). The Canadian National Breast Screening Study-1: breast cancer mortality after 11 to 16 years of follow-up. A randomized screening trial of mammography in women age 40 to 49 years.. Ann Intern Med.

[3] Zahl PH, Strand BH, Maehlen J (2004). Incidence of breast cancer in Norway and Sweden during introduction of nationwide screening: prospective cohort study.. Bmj.

[4] Elmore JG, Barton MB, Moceri VM, Polk S, Arena PJ (1998). Ten-year risk of false positive screening mammograms and clinical breast examinations.. N Engl J Med.

[5] Rosenberg RD, Hunt WC, Williamson MR, Gilliland FD, Wiest PW (1998). Effects of age, breast density, ethnicity, and estrogen replacement therapy on screening mammographic sensitivity and cancer stage at diagnosis: review of 183,134 screening mammograms in Albuquerque, New Mexico.. Radiology.

[6] Kerlikowske K, Grady D, Barclay J, Sickles EA, Ernster V (1996). Likelihood ratios for modern screening mammography. Risk of breast cancer based on age and mammographic interpretation.. Jama.

[7] Kriege M, Brekelmans CT, Boetes C, Besnard PE, Zonderland HM (2004). Efficacy of MRI and mammography for breast-cancer screening in women with a familial or genetic predisposition.. N Engl J Med.

[8] Miki Y, Swensen J, Shattuck-Eidens D, Futreal PA, Harshman K (1994). A strong candidate for the breast and ovarian cancer susceptibility gene BRCA1.. Science.

[9] Wooster R, Bignell G, Lancaster J, Swift S, Seal S (1995). Identification of the breast cancer susceptibility gene BRCA2.. Nature.

[10] Seal S, Thompson D, Renwick A, Elliott A, Kelly P (2006). Truncating mutations in the Fanconi anemia J gene BRIP1 are low-penetrance breast cancer susceptibility alleles.. Nat Genet.

[11] Weischer M, Bojesen SE, Tybjaerg-Hansen A, Axelsson CK, Nordestgaard BG (2007). Increased risk of breast cancer associated with CHEK2*1100delC.. J Clin Oncol.

[12] Renwick A, Thompson D, Seal S, Kelly P, Chagtai T (2006). ATM mutations that cause ataxia-telangiectasia are breast cancer susceptibility alleles.. Nat Genet.

[13] Akashi M, Koeffler HP (1998). Li-Fraumeni syndrome and the role of the p53 tumor suppressor gene in cancer susceptibility.. Clin Obstet Gynecol.

[14] Coles C, Condie A, Chetty U, Steel CM, Evans HJ (1992). p53 mutations in breast cancer.. Cancer Res.

[15] Colditz GA, Willett WC, Hunter DJ, Stampfer MJ, Manson JE (1993). Family history, age, and risk of breast cancer. Prospective data from the Nurses' Health Study.. Jama.

[16] Slattery ML, Kerber RA (1993). A comprehensive evaluation of family history and breast cancer risk. The Utah Population Database.. Jama.

[17] Johnson N, Lancaster T, Fuller A, Hodgson SV (1995). The prevalence of a family history of cancer in general practice.. Fam Pract.

[18] Sjoblom T, Jones S, Wood LD, Parsons DW, Lin J (2006). The consensus coding sequences of human breast and colorectal cancers.. Science.

[19] Jones PA, Baylin SB (2002). The fundamental role of epigenetic events in cancer.. Nat Rev Genet.

[20] Ting AH, McGarvey KM, Baylin SB (2006). The cancer epigenome–components and functional correlates.. Genes Dev.

[21] Feinberg AP, Ohlsson R, Henikoff S (2006). The epigenetic progenitor origin of human cancer.. Nat Rev Genet.

[22] Miyamoto K, Ushijima T (2005). Diagnostic and therapeutic applications of epigenetics.. Jpn J Clin Oncol.

[23] Hoque MO, Feng Q, Toure P, Dem A, Critchlow CW (2006). Detection of aberrant methylation of four genes in plasma DNA for the detection of breast cancer.. J Clin Oncol.

[24] Fackler MJ, McVeigh M, Mehrotra J, Blum MA, Lange J (2004). Quantitative multiplex methylation-specific PCR assay for the detection of promoter hypermethylation in multiple genes in breast cancer.. Cancer Res.

[25] Lippman Z, Gendrel AV, Black M, Vaughn MW, Dedhia N (2004). Role of transposable elements in heterochromatin and epigenetic control.. Nature.

[26] Lippman Z, Gendrel AV, Colot V, Martienssen R (2005). Profiling DNA methylation patterns using genomic tiling microarrays.. Nat Methods.

[27] Ordway JM, Bedell JA, Citek RW, Nunberg A, Garrido A (2006). Comprehensive DNA methylation profiling in a human cancer genome identifies novel epigenetic targets.. Carcinogenesis.

[28] Shames DS, Girard L, Gao B, Sato M, Lewis CM (2006). A genome-wide screen for promoter methylation in lung cancer identifies novel methylation markers for multiple malignancies.. PLoS Med.

[29] Korshunova K, Maloney RK, Lakey N, Citek RW, Bacher B Massively Parallel Bisulphite Pyrosequencing Reveals the Molecular Complexity of Breast Cancer Associated Cytosine Methylation Patterns Obtained from Tissue and Serum DNA.. Genome Research..

[30] McKee KK, Palyha OC, Feighner SD, Hreniuk DL, Tan CP (1997). Molecular analysis of rat pituitary and hypothalamic growth hormone secretagogue receptors.. Mol Endocrinol.

[31] Petersenn S, Rasch AC, Penshorn M, Beil FU, Schulte HM (2001). Genomic structure and transcriptional regulation of the human growth hormone secretagogue receptor.. Endocrinology.

[32] Jeffery PL, Herington AC, Chopin LK (2003). The potential autocrine/paracrine roles of ghrelin and its receptor in hormone-dependent cancer.. Cytokine Growth Factor Rev.

[33] Jeffery PL, Murray RE, Yeh AH, McNamara JF, Duncan RP (2005). Expression and function of the ghrelin axis, including a novel preproghrelin isoform, in human breast cancer tissues and cell lines.. Endocr Relat Cancer.

[34] Barzon L, Pacenti M, Masi G, Stefani AL, Fincati K (2005). Loss of growth hormone secretagogue receptor 1a and overexpression of type 1b receptor transcripts in human adrenocortical tumors.. Oncology.

[35] Howard AD, Feighner SD, Cully DF, Arena JP, Liberator PA (1996). A receptor in pituitary and hypothalamus that functions in growth hormone release.. Science.

[36] Deming SL, Nass SJ, Dickson RB, Trock BJ (2000). C-myc amplification in breast cancer: a meta-analysis of its occurrence and prognostic relevance.. Br J Cancer.

[37] Linke SP, Bremer TM, Herold CD, Sauter G, Diamond C (2006). A multimarker model to predict outcome in tamoxifen-treated breast cancer patients.. Clin Cancer Res.

[38] Scorilas A, Trangas T, Yotis J, Pateras C, Talieri M (1999). Determination of c-myc amplification and overexpression in breast cancer patients: evaluation of its prognostic value against c-erbB-2, cathepsin-D and clinicopathological characteristics using univariate and multivariate analysis.. Br J Cancer.

[39] Naidu R, Wahab NA, Yadav M, Kutty MK (2002). Protein expression and molecular analysis of c-myc gene in primary breast carcinomas using immunohistochemistry and differential polymerase chain reaction.. Int J Mol Med.

[40] Chrzan P, Skokowski J, Karmolinski A, Pawelczyk T (2001). Amplification of c-myc gene and overexpression of c-Myc protein in breast cancer and adjacent non-neoplastic tissue.. Clin Biochem.

[41] Grandori C, Cowley SM, James LP, Eisenman RN (2000). The Myc/Max/Mad network and the transcriptional control of cell behavior.. Annu Rev Cell Dev Biol.

[42] Ogawa H, Ishiguro K, Gaubatz S, Livingston DM, Nakatani Y (2002). A complex with chromatin modifiers that occupies E2F- and Myc-responsive genes in G0 cells.. Science.

[43] Hurlin PJ, Huang J (2006). The MAX-interacting transcription factor network.. Semin Cancer Biol.

[44] Yang YH, Dudoit S, Luu P, Lin DM, Peng V (2002). Normalization for cDNA microarray data: a robust composite method addressing single and multiple slide systematic variation.. Nucleic Acids Res.

[45] Wolfinger RD, Gibson G, Wolfinger ED, Bennett L, Hamadeh H (2001). Assessing gene significance from cDNA microarray expression data via mixed models.. J Comput Biol.

[46] Hochberg Y, Benjamini Y (1990). More powerful procedures for multiple significance testing.. Stat Med.

[47] Choi YC, Chae CB (1991). DNA hypomethylation and germ cell-specific expression of testis-specific H2B histone gene.. J Biol Chem.

[48] Frommer M, McDonald LE, Millar DS, Collis CM, Watt F (1992). A genomic sequencing protocol that yields a positive display of 5-methylcytosine residues in individual DNA strands.. Proc Natl Acad Sci U S A.

[49] Ordway JM, Bedell JA, Citek RW, Nunberg AN, Jeddeloh JA (2005). MethylMapper: a method for high-throughput, multilocus bisulfite sequence analysis and reporting.. Biotechniques.

