# Supplementary material for: Identification of Novel High-Frequency DNA Methylation Changes in Breast Cancer
Source: PLoS One. 2007 Dec 19;2(12):e1314. doi: 10.1371/journal.pone.0001314 (PMC2117343; doi:10.1371/journal.pone.0001314)
Supplement: Figure S1 — Repeated GHSR qPCR analyses for samples near the 0.64 threshold for sensitivity and specificity calculations. 16 samples were analyzed three times (REP1, REP2, REP3). Digestions were performed using a different lot of McrBC enzyme than that used for the experiments summarized in Table 1 (ORIGINAL). Each value is an averaged dCt between two qPCR technical replicates. Samples scoring above the threshold are indicated in red, and those scoring below the threshold are indicated in green. (0.01 MB PDF) [file pone.0001314.s001.pdf]

Figure S1.

	ORIGINAL	REP1	REP2	REP3
TUMOR	0.75	0.755	0.905	1.12
TUMOR	0.835	2.67	2.53	2.63
TUMOR	0.915	2.255	1.715	1.65
TUMOR	1.115	1.265	1.27	1.325
TUMOR	1.125	1.565	1.59	1.585
TUMOR	1.13	1.495	1.54	1.565
TUMOR	1.22	1.23	1.375	1.295
TUMOR	1.225	1.84	1.625	1.665
TUMOR	1.41	1.895	1.705	1.59
TUMOR	1.5	1.71	1.7	1.61
TUMOR	1.335	1.5	1.46	1.47
NORMAL	0.545	0	0	0
NORMAL	0.71	0.03	0	0.1
NORMAL	0.805	0.305	0.275	0.33
NORMAL	0.805	0	0	0.25
NORMAL	0.525	0.46	0.55	0.61
