# Supplementary figures and images for: Identification of Novel High-Frequency DNA Methylation Changes in Breast Cancer
Source: PLoS One. 2007 Dec 19;2(12):e1314. doi: 10.1371/journal.pone.0001314 (PMC2117343; doi:10.1371/journal.pone.0001314)

**Figure S2.**

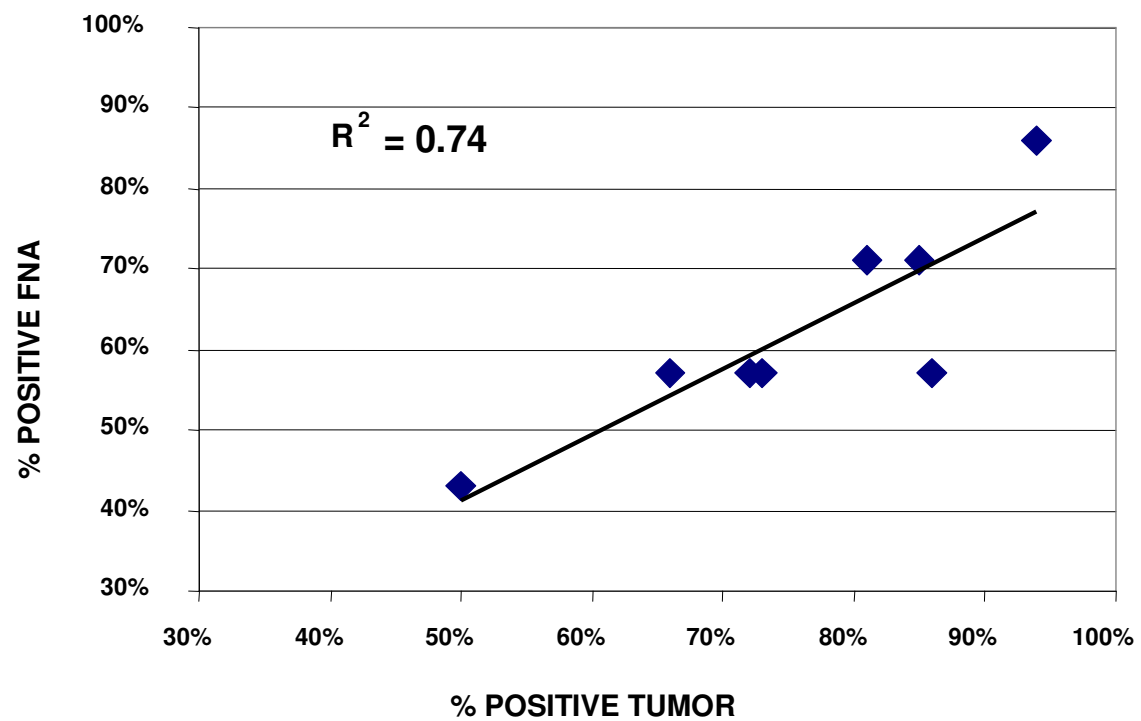

Supplement: Figure S2 — Detection of tumor-specific DNA hypermethylation in fine needle aspirate specimens. Eight biomarker loci were screened in seven FNA samples obtained from confirmed breast cancer cases. For each locus, the percentage of FNA samples that reported hypermethylation was plotted against the percentage of independent tumor samples that reported hypermethylation. If the biomarkers are detecting breast cancer at the same frequency as in tissue samples the expectation is that the two results should be directly proportional (i.e. exhibit a sensitivity slope of 1.0). This theoretical maxim is indicated by the dashed y = x line. The actual slope (solid line) and its R2 are indicated. The theoretical and experimental results are not significantly different (n = 8 data points). (0.01 MB PDF) [file pone.0001314.s002.pdf]

Figure S3.

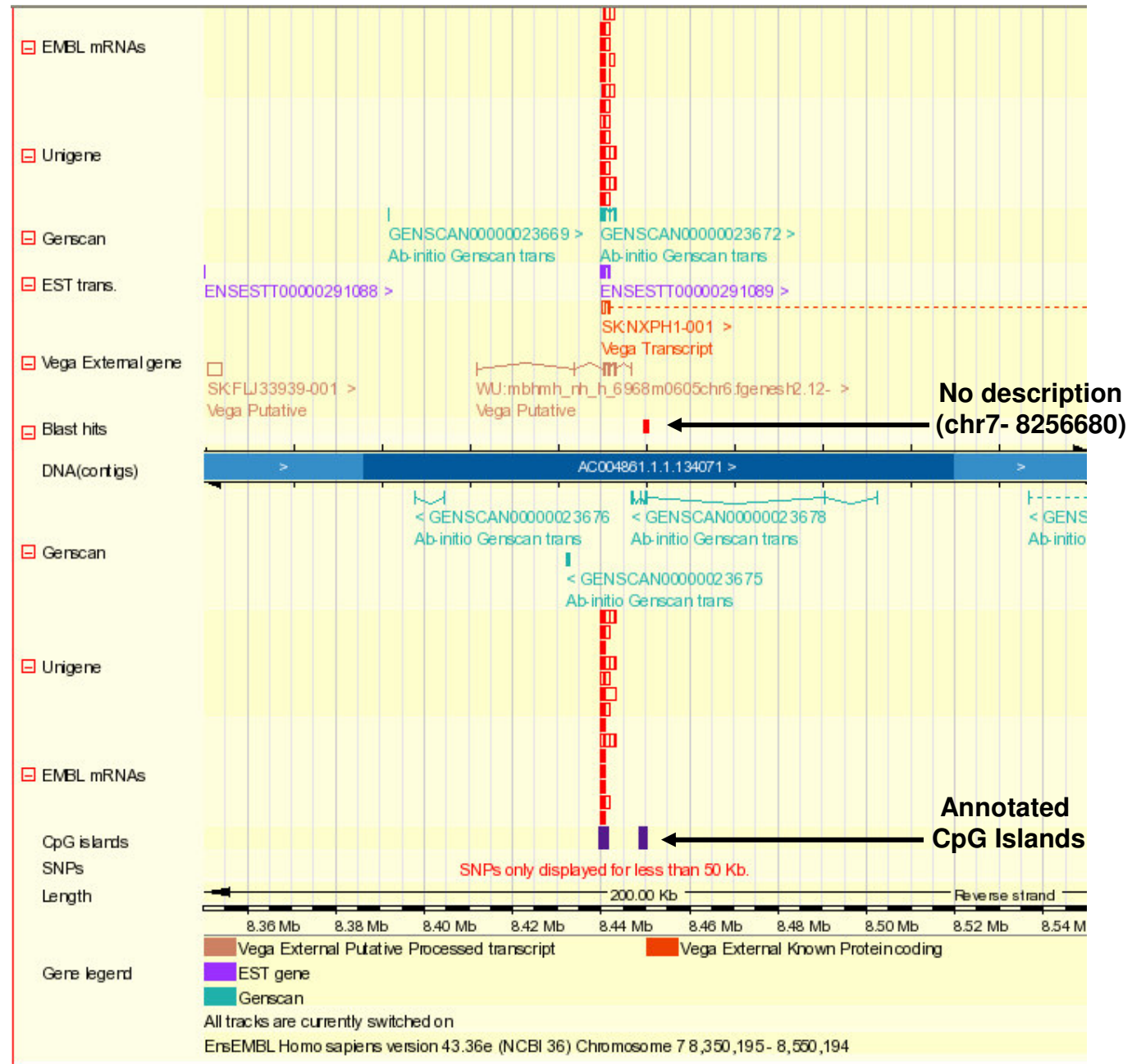

Supplement: Figure S3 — Ensembl contig view of the uncharacterized locus corresponding to chr7-8256680(NCBI35). The position of the microarray feature that reported differential DNA methylation and Ensembl annotated CpG islands are indicated by arrows (NCBI36(hg18)). (0.16 MB PDF) [file pone.0001314.s003.pdf]
