# Supplementary material for: Identification of Novel High-Frequency DNA Methylation Changes in Breast Cancer
Source: PLoS One. 2007 Dec 19;2(12):e1314. doi: 10.1371/journal.pone.0001314 (PMC2117343; doi:10.1371/journal.pone.0001314)
Supplement: Supplementary file 3 [file pone.0001314.s004.doc]

## Sheet: Discovery Panel

Supplemental Table 1.  Patient demographics for samples used in microarray analyses.
Matched Pair ID	sample_id	GCI_DIAGNOSIS	TUMOR_PERCENT	WEIGHT (GRAMS)	age	sex	BMI	TOBACCO	DRINK_ALCOHOL	DIAGNOSIS_AGE	MENOPAUSAL_STATUS	HORMONE_REPLACEMENT	CANCER_STAGE	CANCER_SUBCLASS	LYMPH_NODES	RECEPTORS	CANCERS	CANCER_YOU	CANCER_FATHER	CANCER_MOTHER	CANCER_ANYBROTHER	CANCER_ANYSISTER	CANCER_FATHERFATHER	CANCER_FATHERMOTHER	CANCER_MOTHERMOTHER	CANCER_MOTHERFATHER
1	1	INFILTRATING DUCTAL CARCINOMA	70	1.13	38	FEMALE	22.1	NO	NO	38	PREMENOPAUSAL	NO	STAGE II	A	0	BOTH	BREAST CANCER	X
1	2	ADJACENT NORMAL BREAST	0	1.7	.		.			.					.		NONE OF THE ABOVE CANCER		X	X	X	X	X	X	X	X
2	3	INFILTRATING DUCTAL CARCINOMA	65	1.85	42	FEMALE	16.4	NO	NO	42	PREMENOPAUSAL	NO	STAGE II	A	0	BOTH	BREAST CANCER	X
2	4	ADJACENT NORMAL BREAST	0	1.82	.		.			.					.		NONE OF THE ABOVE CANCER		X	X	X	X	X	X	X	X
3	5	INFILTRATING DUCTAL CARCINOMA	70	2.21	57	FEMALE	23.4	NO	NO	57	POST MENOPAUSAL	NO	STAGE II	A	0	BOTH	BREAST CANCER	X
3	6	ADJACENT NORMAL BREAST	0	1.99	.		.			.					.		NONE OF THE ABOVE CANCER		X	X	X	X	X	X	X	X
4	7	INFILTRATING DUCTAL CARCINOMA	80	2.04	46	FEMALE	23.6	NO	NO	46	PREMENOPAUSAL	NO	STAGE II	B	7	NO	BREAST CANCER	X
4	8	ADJACENT NORMAL BREAST	0	1.8	.		.			.					.		NONE OF THE ABOVE CANCER		X	X	X	X	X	X	X	X
5	9	INFILTRATING DUCTAL CARCINOMA	95	1.36	34	FEMALE	18	NO	NO	34	PREMENOPAUSAL	NO	STAGE II	B	1	NO	BREAST CANCER	X
5	10	ADJACENT NORMAL BREAST	0	1.66	.		.			.					.		NONE OF THE ABOVE CANCER		X	X	X	X	X	X	X	X
6	11	INFILTRATING DUCTAL CARCINOMA	70	1.98	41	FEMALE	20.4	NO	NO	40	PREMENOPAUSAL	NO	STAGE II	B	0	NO	BREAST CANCER	X
6	12	ADJACENT NORMAL BREAST	0	1.96	.		.			.					.		NONE OF THE ABOVE CANCER		X	X	X	X	X	X	X	X
7	13	INFILTRATING DUCTAL CARCINOMA	75	1.82	52	FEMALE	20.3	NO	NO	52	PERIMENOPAUSAL	NO	STAGE II	A	0	NO	BREAST CANCER	X
7	14	ADJACENT NORMAL BREAST	0	1.82	.		.			.					.		NONE OF THE ABOVE CANCER		X	X	X	X	X	X	X	X
8	15	INFILTRATING DUCTAL CARCINOMA	80	2.01	47	FEMALE	22.6	NO	NO	47	PERIMENOPAUSAL	NO	STAGE II	A	0	NO	BREAST CANCER	X
8	16	ADJACENT NORMAL BREAST	0	2.23	.		.			.					.		NONE OF THE ABOVE CANCER		X	X	X	X	X	X	X	X
9	17	INFILTRATING DUCTAL CARCINOMA	70	1.59	48	FEMALE	22	NO	NO	48	PREMENOPAUSAL	NO	STAGE II	A	3	NO	BREAST CANCER	X
9	18	ADJACENT NORMAL BREAST	0	1.42	.		.			.					.		NONE OF THE ABOVE CANCER		X	X	X	X	X	X	X	X

## Sheet: Legend

DCM Name	Question Group Name	Question Name	Description
Matched Pair ID			ID matches those indicated in Figure 1
DEMOG	DEMOGRAPHY	SAMPLE_ID	Sample ID
		BIRTH_YEAR	Applicant Birth Year
		AGE	Applicant Age
		SEX	Sex
		BMI	Body Mass Index
		DCMDATE	Date of Collection of Material
		HEIGHT	Height
		HEIGHT_UNIT	Height Units
		HEIGHT_CM_DER	Derive Height in Centimeters
		WEIGHT	Weight
		WEIGHT_UNIT	Weight Units
		WEIGHT_KG_DER	Derive Weight in Kilograms
	FOOD	VEGETABLESPERDAY	Servings of Vegetables / Day
		FRUITSPERDAY	Servings of Fruits / Day
		GRAINSPERDAY	Servings of Grains / Day
		DAIRYPERDAY	Servings of Dairy / Day
		MEATPERWEEK	Servings of Meat / Week
		FISHPERWEEK	Servings of Fish / Week
	EXERCISE	EXERCISEPERWEEK	Times of Exercise / Week
		EXERCISEMINUTES	Minutes exercised each time
	ALCOHOL_TOBACCO	TOBACCO	Tobacco Use Y/N
		TOBACCO_PERDAY	Tobacco Use / Day
		TOBACCO_TYPE	Type of Tobacco
		TOBACCO_TYPE_OTHER	Other Type of Tobacco
		TOBACCO_TYPE_MULTI	For Multiple TOBACCO Types
		TOBACCO_USEYEARS	Number of Use Years
		TOBACCO_CURRENT	Current User of Tobacco
		TOBACCO_QUITYEARS	Number of years quit
		DRINK_ALCOHOL	Drink Alcohol Y/N
		DRINKS_NUMBER	Number of Drinks / week
	OCCUPATION	OCCUPATION	Occupation Verbatim Term
		OCC_DETAILED_TERM	Occupation Detailed Term derived from TMS
		OCC_MAJOR_GRP_TERM	Occupation Major Group Term derived from
		OCC_MINOR_GRP_TERM	Occupation Minor Group Term
ETHNICITY	ETHNICITY	ETHNIC_BACKGROUND	Ethnic Background
		ETHNIC_YOU	Applicants Ethnicity
		ETHNIC_FATHER	Fathers Ethnicity
		ETHNIC_MOTHER	Mothers Ethnicity
		ETHNIC_FATHERFATHER	Fathers Fathers Ethnicity
		ETHNIC_FATHERMOTHER	Fathers Mothers Ethnicity
		ETHNIC_MOTHERFATHER	Mothers Fathers Ethnicity
		ETHNIC_MOTHERMOTHER	Mothers Mothers Ethnicity
CANCER_HIST	CANCER_HISTORY	CANCERS	Cancer
		CANCER_YOU	Applicant's Cancer
		CANCER_FATHER	Fathers Cancer
		CANCER_MOTHER	Mothers Cancer
		CANCER_ANYBROTHER	Any Brothers Cancer
		CANCER_ANYSISTER	Any Sisters Cancer
		CANCER_FATHERFATHER	Fathers Father Cancer
		CANCER_FATHERMOTHER	Fathers Mother Cancer
		CANCER_MOTHERMOTHER	Mothers Mother Cancer
		CANCER_MOTHERFATHER	Mothers Father Cancer
MED_HIST	MEDICAL_HISTORY	DISEASE	Disease Verbatim Term
		DISEASE_YOU	Applicant Disease
		DISEASE_FATHER	Fathers Disease
		DISEASE_MOTHER	Mothers Disease
		DISEASE_ANYBROTHER	Any Brothers Disease
		DISEASE_ANYSISTER	Any Sisters Disease
		DISEASE_FATHERFATHER	Fathers Father Disease
		DISEASE_FATHERMOTHER	Fathers Mother Disease
		DISEASE_MOTHERMOTHER	Mothers Mother Disease
		DISEASE_MOTHERFATHER	Mothers Father Disease
MED_HIST_OTHER	MEDICAL_HIST_OTHR	DISEASE_OTHER	Other Disease
		DO_MAIN_TERM	Disease Other Main Term derived from TMS
		DO_HIGH_LVL_BODY	Disease Other High Level Body System derived from TMS
		DO_SUB_TERM	Disease Other Sub Term derived from TMS
		OTHER_YOU	Applicant Other
ADVERSE_EVENTS	PRESCIPTION_DISCONT	REMOVED_FROM_DRUG	Removed From Drug
	ADVERSE_EVENTS	ADVERSE_REACTION	Adverse Reaction Verbatim Term
		AE_PT_TERM	AE Preferred Term derived from TMS
		AE_LL_TERM	AE Low Level Term derived from TMS
		AE_SOC_TERM	AE System Organ Class Term derived from TMS
		AED_NAME	AE Drug Name
		AED_PREF_TERM	AE Drug Preferred Term derived from TMS
		AED_ATC1_TERM	AE Drug ATC Level 1 Term derived from TMS
		AED_ATC3_TERM	AE Drug ATC Level 3 Term
MEDICATIONS	MEDS_NONE	DRUG_NONE	Taking any prescription meds?
	MEDICATIONS	DRUG_TYPE	Drug type (p=prescription, c=over-the-counter, s=supplement)
		DRUG_NAME	Drug Name
		DRUG_PREF_TERM	Drug Preferred Term derived from TMS
		DRUG_ATC1_TERM	Drug ATC Level 1 Term derived from TMS
		DRUG_ATC3_TERM	Drug ATC Level 3 Term
		DRUG_STARTYEAR	Drug Started Year
BREAST_CANCER_1	BC_SIGNS	LUMP_BREAST	Lump in breast
		ULCERATION_BREAST	Ulceration in breast
		PAIN_TENDER_BREAST	Pain or tenderness in breast
		REDNESS_BREAST	Redness on breast
		LUMP_ARMPIT	Lump in armpit
		SWELLING_BREAST	Swelling of the breast
		NIPPLE_DISCHARGE	Nipple discharge
		DIMPLING_BREAST	Dimpling breast
		NIPPLE_SORENESS	Nipple Soreness
		NIPPLE_DISCOLOR	Nipple discoloration
		NIPPLE_RETRACTION	Nipple Retraction
		OTHER_SYM	Other symptom prior to diagnosis?
		OTHER_SYM_SPECIFY	Other Symptom Specify
		ASSYMPTOMATIC	Assymptomatic
	BC_DIAGNOSIS	DIAGNOSIS_AGE	Age diagnosed with Breast Cancer
		INDICATIONS_BC	First indication of Breast Cancer
		INDICATION_SPECIFY	Indications Specify
		SELF_EXAM	Prior to dx self examinations
		MAMMOGRAPHY	Prior to dx mammography
		CHECK_UPS	Prior to dx check-ups (how often)
	BC_HISTORY	BENIGN	History of benign breast disease?
		BREASTDISEASE_TYPE	Type of breast disease
BREAST_CANCER_2	BIRTH_HISTORY	RADIATION_EXPOSURE	Have they had radiation exposure
		MENSTRUATE_AGE	Age of 1st menstruation
		PREG_TIMES	How many times pregnant
		PREG_TOTERM	How many pregnancies made it to term
		AGE_AT_FIRST_CHILD	How old was she at the birth of child 1
		BREASTFEED_CHILD	how many children did she breast feed
	MENOPAUSE	ORAL_CONTRACEPTIVES	Ever used oral contraceptives?
		ORAL_CON_LENGTH	Total length of time of oral contracepti
		ORAL_CON_UNIT	Unit for time (months or years)
		ORAL_CON_DK	Don't know how long on oral con
		HYSTERECTOMY	has she ever had a hysterectomy
		HYST_PROCEDURE	Surgical procedure for hysterctomy
		MENOPAUSAL_STATUS	Menopausal status
		LAST_PERIOD	When was last period
		MENOPAUSE_AGE	Unit (age or years ago)
		LAST_PERIOD_DK	Don't know when last period was
BREAST_CANCER_3	MENOPAUSE	HORMONE_REPLACEMENT	Ever used HRT to control menopausal symp
		HORMONE_LENGTH	Total length of time of HRT use?
		HORMONE_UNIT	Unit for time (months or years)
		HORMONE_DK	Don't know how long HRT used for
		HORMONE_CURRENT	Currently use hormones?
	BC_TYPE	DUCTAL	Ductal carcinoma in situ
		MUCINOUS_COLLOID	Mucinous/colloid carcinoma
		LOBULAR	Lobular carcinoma in situ
		INFILTRATING_LOBULAR	Infiltrating lobular carcinoma
		INFILTRATING_DUCTAL	Infiltrating ductal carcinoma
		INFLAMMATORY	inflammatory
		MEDULLARY	medullary
		PAGETS	pagets
	CANCER_STAGE	CANCER_STAGE	breast cancer stage
		CANCER_SUBCLASS	Cancer subclass
	CANCER_LOCATION	BC_LOCATION	breast cancer location
		LOCATION_SPECIFY	location specify
		LYMPH_NODES	lymph nodes
		LYMPH_NODES_DK	lymph nodes dont know
		RECEPTORS	Reports if receptors are positive to estrogen, progesterone, both, or neither
		HER_2_NEU_IMMUNO	HER-2/neu immunohistochemistry test result
		HER_2_NEU_FISH	HER-2/neu FISH test result
		HER_2_NEU_CHRM_RATIO	HER-2/neu Chromosome 17 Ratio
BREAST_CANCER_4	THERAPIES	CHEMOTHERAPY	chemotherapy
		RADIATION	radiation
		SURGERY	Surgery
		HORMONAL	hormonal
		RADIATION_TYPE	radiation type
		RADIATION_TYPE_OTHER	radiation type other
	BC_SURGERY	RADICALMASTECTOMY	radical mastectomy
		SIMPLEMASTECTOMY	simple mastectomy
		MODIFIEDRADICAL	modified radical
		LUMPECTOMY	lumpectomy
	HORMONE_THERAPY_2	TAMOXIFEN	tamoxifen
		AMINOGLUTETHIMIDE	aminog
		TOREMIFENE	toremifene
		LEUPROLIDE	leuprolide
		ANASTROZOLE	anastrozole
		GOSERELIN	goserelin
		LETROZOLE	letrozole
		MEGESTROL	megestrol
		OTHER	Symptoms - other
		OTHER_SPECIFY	Other, specify
		HORMONE_LENGTH_4	Total length of time of hormone use
		HORMONE_UNIT_4	months or years
		HORMONE_DK_4	Total length of time of hormone use
		TREATMENT_RESPONSE	treatment response
BREAST_CANCER_5	RELAPSE1	RELAPSE	relapse
		AFTERTREATMENT	how many yrs after tx was relapse
		AFTERTREATMENT_DK	don't know
		RELAPSE_LOCATION	relapse location
		RELAPSE_SPECIFY	relapse specify
		CHEMOTHERAPY1	chemotherapy
		RADIATION1	radiation
		SURGERY1	Surgery
		HORMONAL1	hormonal
		RELAPSE_RESPONSE	relapse response
	REMISSION	REMISS_LENGTH	Remission Length
		REMISS_LENGTH_UNIT	remission length (month/year)
		REMISS_LENGTH_DK	remission length dont know
	BONE_METASTASES	PAMIDRONATE	pamidronate
		BM_TRANSPLANT	bone marrow transplant
		BM_LENGTH	how long ago was transplant
		BM_LENGTH_DK	bm length dont know
	CURRENT_STATUS	CURRENT_STATUS	current status
CANCER_REPORTS	CANCER_REPORTS	PATHOLOGY_RPTC	Pathology Report
		LABS_ARPT	Labs Reports
		OTHER_ARPT	Other Report Attached Report
 	.
