# Supplementary material for: Identification of Novel High-Frequency DNA Methylation Changes in Breast Cancer
Source: PLoS One. 2007 Dec 19;2(12):e1314. doi: 10.1371/journal.pone.0001314 (PMC2117343; doi:10.1371/journal.pone.0001314)
Supplement: Supplementary file 4 [file pone.0001314.s007.doc]

## Sheet: sheet 1

Supplemental Table 4.  Patient demographics for samples used in validation analyses.
TUMOR SAMPLES
TISSUE_ID	GCI_DIAGNOSIS	TUMOR_PERCENT	WEIGHT (GRAMS)	age	sex	BMI	TOBACCO	TOBACCO_PERDAY	TOBACCO_TYPE	TOBACCO_USEYEARS	TOBACCO_CURRENT	TOBACCO_QUITYEARS	DRINK_ALCOHOL	DRINKS_NUMBER	MENOPAUSAL_STATUS	HORMONE_REPLACEMENT	HORMONE_LENGTH	HORMONE_UNIT	CANCER_STAGE	CANCER_SUBCLASS	LYMPH_NODES	RECEPTORS	RELAPSE	RELAPSE_LOCATION	CANCERS	CANCER_YOU	CANCER_FATHER	CANCER_MOTHER	CANCER_ANYBROTHER	CANCER_ANYSISTER	CANCER_FATHERFATHER	CANCER_FATHERMOTHER	CANCER_MOTHERMOTHER	CANCER_MOTHERFATHER
1	INFILTRATING DUCTAL CARCINOMA	65	0.3	88	FEMALE	29.1	NO	.		.		.	NO	.	POST MENOPAUSAL	NO	.		STAGE II	A	0	DONT KNOW	NO		BREAST CANCER	X
2	INFILTRATING DUCTAL CARCINOMA	65	0.35	65	FEMALE	26.1	NO	.		.		.	NO	0	POST MENOPAUSAL	NO	.		STAGE I		0	NO	YES	SECOND PRIMARY SITE	BREAST CANCER	X
3	INFILTRATING DUCTAL CARCINOMA	60	0.74	74	FEMALE	40	YES	20	CIGARETTES	50	YES	.	NO	.	POST MENOPAUSAL	YES	25	YEARS	STAGE II	B	0	DONT KNOW	NO		BREAST CANCER	X
4	INFILTRATING DUCTAL CARCINOMA	70	0.51	29	FEMALE	35.9	NO	.		.		.	YES	1	PREMENOPAUSAL	NO	.		STAGE II	A	.	NO	NO		BREAST CANCER	X		X
5	INFILTRATING DUCTAL CARCINOMA	80	0.47	70	FEMALE	30	YES	10	CIGARETTES	3	NO	20	NO	.	PERIMENOPAUSAL	NO	.		STAGE II	A	.	DONT KNOW	NO		BREAST CANCER	X				X		X
6	INFILTRATING DUCTAL CARCINOMA	75	0.42	87	FEMALE	25	NO	.		.		.	NO	.	POST MENOPAUSAL	NO	.		STAGE I		0	DONT KNOW	NO		BREAST CANCER	X
7	INFILTRATING DUCTAL CARCINOMA	75	0.86	53	FEMALE	50.3	NO	.		.		.	NO	.	POST MENOPAUSAL	NO	.		STAGE II	A	.	NO	NO		BREAST CANCER	X
8	INFILTRATING DUCTAL CARCINOMA	60	1.12	32	FEMALE	30.3	NO	.		.		.	NO	.	PREMENOPAUSAL	NO	.		STAGE II	B	0	DONT KNOW	NO		BREAST CANCER	X								X
9	INFILTRATING DUCTAL CARCINOMA	65	0.58	31	FEMALE	21.8	NO	.		.		.	NO	.	PREMENOPAUSAL	NO	.		STAGE II	A	.	DONT KNOW	NO		BREAST CANCER	X							X
10	INFILTRATING DUCTAL CARCINOMA	60	0.29	39	FEMALE	28	NO	.		.		.	NO	.	PREMENOPAUSAL	YES	9	YEARS	STAGE II	A	0	DONT KNOW	NO		BREAST CANCER	X
11	INFILTRATING DUCTAL CARCINOMA	60	0.26	82	FEMALE	23.9	YES	4	CIGARETTES	6	NO	50	NO	.	POST MENOPAUSAL	NO	.		STAGE II	B	1	ESTROGEN	NO		BREAST CANCER	X
12	INFILTRATING DUCTAL CARCINOMA	85	0.56	35	FEMALE	24.7	NO	.		.		.	NO	.	PREMENOPAUSAL	NO	.		STAGE I		0	NO	NO		BREAST CANCER	X		X
13	INFILTRATING DUCTAL CARCINOMA	85	0.25	74	FEMALE	26.6	YES	20	CIGARETTES	40	NO	12	YES	6	POST MENOPAUSAL	YES	6	MONTHS	STAGE II	B	1	ESTROGEN	NO		BREAST CANCER	X
14	INFILTRATING DUCTAL CARCINOMA	65	0.28	82	FEMALE	32.9	YES	5	CIGARETTES	25	NO	50	NO	.	POST MENOPAUSAL	YES	25	YEARS	STAGE II	A	0	DONT KNOW			BREAST CANCER	X
15	INFILTRATING DUCTAL CARCINOMA	80	1.03	77	FEMALE	49.1	YES	40	CIGARETTES	40	NO	16	NO	.	POST MENOPAUSAL	NO	.		STAGE II	A	0	NO	NO		BREAST CANCER	X
16	INFILTRATING DUCTAL CARCINOMA	90	0.7	56	FEMALE	24.6	NO	.		.		.	NO	.	POST MENOPAUSAL	NO	.		STAGE II	B	0				BREAST CANCER	X
17	INFILTRATING DUCTAL CARCINOMA	60	0.29	49	FEMALE	24.4	YES	10	CIGARETTES	32	YES	.	NO	.	PERIMENOPAUSAL	NO	.		STAGE II	B	.	DONT KNOW	YES	METASTATIC SITE(S) (SPECIFY)	BREAST CANCER	X
18	INFILTRATING DUCTAL CARCINOMA	65	0.27	60	FEMALE	36.6	NO	.		.		.	NO	.	POST MENOPAUSAL	YES	8	YEARS	STAGE II	A	0	NO	NO		BREAST CANCER	X
19	INFILTRATING DUCTAL CARCINOMA	70	0.53	41	FEMALE	33.1	NO	.		.		.	NO	.	PREMENOPAUSAL	NO	.		STAGE II	B	2	NO	NO		BREAST CANCER	X
20	INFILTRATING DUCTAL CARCINOMA	100	0.26	86	FEMALE	31.3	NO	.		.		.	NO	.	POST MENOPAUSAL	YES	2	YEARS	STAGE II	A	0	NO	NO		BREAST CANCER	X				X
21	INFILTRATING DUCTAL CARCINOMA	70	0.66	33	FEMALE	25.8	NO	.		.		.	NO	.	PREMENOPAUSAL	NO	.		STAGE II	A	0	BOTH	NO		BREAST CANCER	X
22	INFILTRATING DUCTAL CARCINOMA	85	0.31	50	FEMALE	19	NO	.		.		.	NO	.	PREMENOPAUSAL	NO	.		STAGE II	A	0	BOTH	NO		BREAST CANCER	X
23	INFILTRATING DUCTAL CARCINOMA	70	0.95	53	FEMALE	31.9	YES	20	CIGARETTES	30	NO	4	NO	.	PERIMENOPAUSAL	NO	.		STAGE II	B	2	ESTROGEN	NO		BREAST CANCER	X		X		X
24	INFILTRATING DUCTAL CARCINOMA	70	0.37	45	FEMALE	30.8	NO	.		.		.	YES	6	PREMENOPAUSAL	NO	.		STAGE II	A	3	BOTH	NO		BREAST CANCER	X						X
25	INFILTRATING DUCTAL CARCINOMA	60	0.4	69	FEMALE	31.8	YES	30	CIGARETTES	50	YES	.	NO	.	POST MENOPAUSAL	NO	.		STAGE II	B	2	NO	NO		BREAST CANCER	X				X
26	INFILTRATING DUCTAL CARCINOMA	60	1.05	47	FEMALE	43	NO	.		.		.		.	POST MENOPAUSAL	NO	.		STAGE II	B	0	NO	NO		BREAST CANCER	X
27	INFILTRATING DUCTAL CARCINOMA	85	0.35	71	FEMALE	26.2	YES	5	CIGARETTES	5	NO	45	NO	.	POST MENOPAUSAL	NO	.		STAGE II	A	0	NO	NO		BREAST CANCER	X				X			X
28	INFILTRATING DUCTAL CARCINOMA	95	0.3	83	FEMALE	27.5	NO	.		.		.	NO	.	POST MENOPAUSAL	NO	.		STAGE II	A	0	NO	NO		BREAST CANCER	X
29	INFILTRATING DUCTAL CARCINOMA	80	1.66	52	FEMALE	23	NO	.		.		.	NO	.	PERIMENOPAUSAL	NO	.		STAGE II	A	0	NO	NO		BREAST CANCER	X
30	INFILTRATING DUCTAL CARCINOMA	80	1.58	46	FEMALE	26.2	NO	.		.		.	NO	.	PERIMENOPAUSAL	NO	.		STAGE II	A	0	NO	NO		BREAST CANCER	X
31	INFILTRATING DUCTAL CARCINOMA	70	1.57	38	FEMALE	21.1	NO	.		.		.	NO	.	PREMENOPAUSAL	NO	.		STAGE II	A	0	ESTROGEN	NO		BREAST CANCER	X
32	INFILTRATING DUCTAL CARCINOMA	65	1.51	46	FEMALE	23.4	NO	.		.		.	NO	.	PERIMENOPAUSAL	NO	.		STAGE II	A	0	BOTH	NO		BREAST CANCER	X
33	INFILTRATING DUCTAL CARCINOMA	80	1.61	39	FEMALE	26.7	NO	.		.		.	NO	.	PREMENOPAUSAL	NO	.		STAGE II	A	0	ESTROGEN	NO		BREAST CANCER	X
34	INFILTRATING DUCTAL CARCINOMA	60	1.73	39	FEMALE	19.5	NO	.		.		.	NO	.	PREMENOPAUSAL	NO	.		STAGE II	A	0	BOTH	NO		BREAST CANCER	X
35	INFILTRATING DUCTAL CARCINOMA	70	2.05	72	FEMALE	24.8	NO	.		.		.	NO	.	POST MENOPAUSAL	NO	.		STAGE II	B	0	DONT KNOW	NO		BREAST CANCER	X
36	INFILTRATING DUCTAL CARCINOMA	85	1.9	45	FEMALE	22.9	NO	.		.		.	NO	.	PREMENOPAUSAL	NO	.		STAGE II	B	0	DONT KNOW	NO		BREAST CANCER	X
37	INFILTRATING DUCTAL CARCINOMA	65	1.18	38	FEMALE	22.1	NO	.		.		.	NO	.	PREMENOPAUSAL	NO	.		STAGE II	A	0	BOTH	NO		BREAST CANCER	X
38	INFILTRATING DUCTAL CARCINOMA	85	2.08	60	FEMALE	21.6	NO	.		.		.	NO	.	POST MENOPAUSAL	NO	.		STAGE II	A	0	BOTH	NO		BREAST CANCER	X
39	INFILTRATING DUCTAL CARCINOMA	65	1.18	43	FEMALE	19.1	NO	.		.		.	NO	.	PREMENOPAUSAL	NO	.		STAGE II	A	0	NO	NO		BREAST CANCER	X
40	INFILTRATING DUCTAL CARCINOMA	65	1.72	42	FEMALE	16.4	NO	.		.		.	NO	.	PREMENOPAUSAL	NO	.		STAGE II	A	0	BOTH	NO		BREAST CANCER	X
41	INFILTRATING DUCTAL CARCINOMA	70	1.78	40	FEMALE	18.3	NO	.		.		.	NO	.	PREMENOPAUSAL	NO	.		STAGE II	A	0	BOTH	NO		BREAST CANCER	X
42	INFILTRATING DUCTAL CARCINOMA	75	1.82	46	FEMALE	26.3	NO	.		.		.	NO	.	PREMENOPAUSAL	NO	.		STAGE II	B	0	NO	NO		BREAST CANCER	X
43	INFILTRATING DUCTAL CARCINOMA	80	1.36	49	FEMALE	20.5	NO	.		.		.	NO	.	PREMENOPAUSAL	NO	.		STAGE II	A	0	BOTH	NO		BREAST CANCER	X
44	INFILTRATING DUCTAL CARCINOMA	80	1.9	54	FEMALE	21.6	NO	.		.		.	NO	.	PERIMENOPAUSAL	NO	.		STAGE II	B	0	DONT KNOW	NO		BREAST CANCER	X
45	INFILTRATING DUCTAL CARCINOMA	65	1.14	38	FEMALE	15.8	NO	.		.		.	NO	.	PREMENOPAUSAL	NO	.		STAGE I		0	BOTH	NO		BREAST CANCER	X
46	INFILTRATING DUCTAL CARCINOMA	50	1.36	67	FEMALE	18.3	NO	.		.		.	NO	.	POST MENOPAUSAL	NO	.		STAGE I		0	ESTROGEN	NO		BREAST CANCER	X
47	INFILTRATING DUCTAL CARCINOMA	70	1.82	60	FEMALE	22.7	NO	.		.		.	NO	.	POST MENOPAUSAL	NO	.		STAGE II	A	0	BOTH	NO		BREAST CANCER	X
48	INFILTRATING DUCTAL CARCINOMA	60	0.91	50	FEMALE	23.6	NO	.		.		.	NO	.	PREMENOPAUSAL	NO	.		STAGE II	A	0	NO	NO		BREAST CANCER	X
49	INFILTRATING DUCTAL CARCINOMA	60	1.94	73	FEMALE	21.9	NO	.		.		.	NO	.	POST MENOPAUSAL	NO	.		STAGE II	A	0	BOTH	NO		BREAST CANCER	X
50	INFILTRATING DUCTAL CARCINOMA	60	2.12	45	FEMALE	19.4	NO	.		.		.	NO	.	PREMENOPAUSAL	NO	.		STAGE II	A	0	NO	NO		BREAST CANCER	X
51	INFILTRATING DUCTAL CARCINOMA	75	2.08	52	FEMALE	20.3	NO	.		.		.	NO	.	PERIMENOPAUSAL	NO	.		STAGE II	A	0	NO	NO		BREAST CANCER	X
52	INFILTRATING DUCTAL CARCINOMA	75	1.83	47	FEMALE	22.6	NO	.		.		.	NO	.	PERIMENOPAUSAL	NO	.		STAGE II	A	0	NO	NO		BREAST CANCER	X
53	INFILTRATING DUCTAL CARCINOMA	65	1.23	50	FEMALE	21	NO	.		.		.	NO	.	PREMENOPAUSAL	NO	.		STAGE II	A	0	NO	NO		BREAST CANCER	X
54	INFILTRATING DUCTAL CARCINOMA	65	1.53	48	FEMALE	24	NO	.		.		.	NO	.	PERIMENOPAUSAL	NO	.		STAGE II	A	0	PROGESTERONE	NO		BREAST CANCER	X
55	INFILTRATING DUCTAL CARCINOMA	80	1.59	38	FEMALE	19.5	NO	.		.		.	NO	.	PREMENOPAUSAL	NO	.		STAGE II	A	0	DONT KNOW	NO		BREAST CANCER	X
56	INFILTRATING DUCTAL CARCINOMA	70	1.44	42	FEMALE	21.5	NO	.		.		.	NO	.	PREMENOPAUSAL	NO	.		STAGE II	A	0	BOTH	NO		BREAST CANCER	X
57	INFILTRATING DUCTAL CARCINOMA	80	1.93	40	FEMALE	24.2	NO	.		.		.	NO	.	PREMENOPAUSAL	NO	.		STAGE II	A	0	BOTH	NO		BREAST CANCER	X
58	INFILTRATING DUCTAL CARCINOMA	80	1.74	47	FEMALE	24.7	NO	.		.		.	NO	.	PERIMENOPAUSAL	NO	.		STAGE II	A	0	BOTH	NO		BREAST CANCER	X
59	INFILTRATING DUCTAL CARCINOMA	70	1.97	52	FEMALE	20.8	NO	.		.		.	NO	.	POST MENOPAUSAL	NO	.		STAGE II	A	0	NO	NO		BREAST CANCER	X
60	INFILTRATING DUCTAL CARCINOMA	75	1.83	43	FEMALE	18.7	NO	.		.		.	NO	.	PREMENOPAUSAL	NO	.		STAGE II	A	0	DONT KNOW	NO		BREAST CANCER	X
61	INFILTRATING DUCTAL CARCINOMA	65	1.91	72	FEMALE	17.8	NO	.		.		.	NO	.	POST MENOPAUSAL	NO	.		STAGE II	A	0	ESTROGEN	NO		BREAST CANCER	X
62	INFILTRATING DUCTAL CARCINOMA	80	1.87	53	FEMALE	29.6	NO	.		.		.	NO	.	PERIMENOPAUSAL	NO	.		STAGE II	A	0	BOTH	NO		BREAST CANCER	X
63	INFILTRATING DUCTAL CARCINOMA	70	1.77	57	FEMALE	23.4	NO	.		.		.	NO	.	POST MENOPAUSAL	NO	.		STAGE II	A	0	BOTH	NO		BREAST CANCER	X
64	INFILTRATING DUCTAL CARCINOMA	80	1.8	56	FEMALE	20.7	NO	.		.		.	NO	.	POST MENOPAUSAL	NO	.		STAGE II	A	0	NO	NO		BREAST CANCER	X
65	INFILTRATING DUCTAL CARCINOMA	70	1.82	40	FEMALE	19.2	NO	.		.		.	NO	.	PREMENOPAUSAL	NO	.		STAGE II	A	0	DONT KNOW	NO		BREAST CANCER	X
66	INFILTRATING DUCTAL CARCINOMA	85	2.01	60	FEMALE	20.4	NO	.		.		.	NO	.	POST MENOPAUSAL	NO	.		STAGE II	A	0	NO	NO		BREAST CANCER	X
67	INFILTRATING DUCTAL CARCINOMA	90	1.99	41	FEMALE	20.4	NO	.		.		.	NO	.	PREMENOPAUSAL	NO	.		STAGE II	B	0	NO	NO		BREAST CANCER	X
68	INFILTRATING DUCTAL CARCINOMA	90	1.54	58	FEMALE	18.7	NO	.		.		.	NO	.	POST MENOPAUSAL	NO	.		STAGE II	A	0	NO	NO		BREAST CANCER	X
69	INFILTRATING DUCTAL CARCINOMA	90	2.01	39	FEMALE	20.1	NO	.		.		.	NO	.	PREMENOPAUSAL	NO	.		STAGE II	A	0	ESTROGEN	NO		BREAST CANCER	X
70	INFILTRATING DUCTAL CARCINOMA	75	1.45	53	FEMALE	27.8	NO	.		.		.	NO	.	POST MENOPAUSAL	NO	.		STAGE II	A	0	NO	NO		BREAST CANCER	X
71	INFILTRATING DUCTAL CARCINOMA	80	1.39	53	FEMALE	25.1	NO	.		.		.	NO	.	POST MENOPAUSAL	NO	.		STAGE II	A	0	NO	NO		BREAST CANCER	X
72	INFILTRATING DUCTAL CARCINOMA	90	1.76	57	FEMALE	24	NO	.		.		.	NO	.	POST MENOPAUSAL	NO	.		STAGE I		0	DONT KNOW	NO		BREAST CANCER	X
73	INFILTRATING DUCTAL CARCINOMA	70	0.87	55	FEMALE	18.1	NO	.		.		.	NO	.	PERIMENOPAUSAL	NO	.		STAGE II	A	0	DONT KNOW	NO		BREAST CANCER	X
74	INFILTRATING DUCTAL CARCINOMA	65	1.31	36	FEMALE	22.3	NO	.		.		.	NO	.	PREMENOPAUSAL	NO	.		STAGE II	A	0	NO	NO		BREAST CANCER	X
75	INFILTRATING DUCTAL CARCINOMA	85	1.97	51	FEMALE	21.6	NO	.		.		.	NO	.	PERIMENOPAUSAL	NO	.		STAGE I	 	0	DONT KNOW	NO		BREAST CANCER	X
76	INFILTRATING DUCTAL CARCINOMA	65	1.34	58	FEMALE	22.9	NO	.		.		.	NO	.	POST MENOPAUSAL	NO	.		STAGE II	A	0	BOTH	NO		BREAST CANCER	X
77	INFILTRATING DUCTAL CARCINOMA	90	1.96	60	FEMALE	20.1	NO	.		.		.	NO	.	POST MENOPAUSAL	NO	.		STAGE II	A	0	DONT KNOW	NO		BREAST CANCER	X
78	INFILTRATING DUCTAL CARCINOMA	90	1.73	51	FEMALE	23.7	NO	.		.		.	NO	.	PREMENOPAUSAL	NO	.		STAGE II	A	0	NO	NO		BREAST CANCER	X
sample NO	MENOSTTS	MAXDIAM	GRADE	OESTROGE	PROGSTRN	CANCTYPE	LNSTATUS	NO_NODES	RELAPSED	RELDATE	RELSITE
SH5	3	2.2	3	1	3	1	2	0	1
SH19	3	2.7	2	1	3	1	1	1	1
SH29	3	3	3	1	3	1	1	8	2	37343	2
SH30	1	3.7	3	2	3	1	1	1	2	37362	2
SH61	1	1.2	3	1	3	1	2	0	1
SH63	3	2.2	3	1	3	1	2	0	1
SH80		2.7	3	2	3	1	1	4	1
SH100	3	9	3	2	2	1	1	17	1
SH87	1	8.5	3	2	2	1	1	12	1
SH6	1	2.5	2	1	3	1	2	0	1
SH10	1	3	3	1	3	1	1	5	1
SH12	1	2	1	1	3	1	1	1	1
SH16	3	2	3	1	3	1	2	0	1
SH41	3	1.3	3	1	3	1	1	21	1
SH45	1	1.7	2	1	3	1	1	3	1
SH62	3	4.5	3	1	3	1	1	10	2	37252	2
SH65		2.6	2	1	3	1	2	0	1
SH68	3	2.4	2	1	3	1	1	1	1
SH75	3	2	3	1	3	1	1	1	1
SH78	3	4	3	2	3	1	2	0	1
SH79	1	3.2	3	1	3	1	1	17	1
SH70	3	3.5	3	2	2	1	2	0	1
SH84	3	3.5	3	1	1	1	1	1	1
SH89	1	2.8	3	1	1	1	1	8	1
SH99	3	2.8	3	2	2	1	1	1	1
MENOSTTS	- Menopausal status (1=Pre; 2=Post; 3=Peri)
MAXDIAM	- Maximum tumour diameter (cm)
GRADE	- Tumour grade (1=Well; 2=Moderate; 3=Poor; 4=Unknown)
OESTROGE  	- Oestrogen receptors (1=+ve;2=-ve;3=Unknown)
PROGSTRN	- Progesterone receptors (1=+ve;2=-ve;3=Unknown)
CANCTYPE	- Type of invasive cancer (1=Ductal;2=Lobular; 3=Other)
LNSTATUS	- LN status (1=+ve; 2=-ve; 3=Unknown)
NO_NODES  	- Number of nodes involved
RELAPSED	- Has patient relapsed (1=No; 2=Yes)
RELDATE	- Date of relapse
RELSITE	- Site of relapse (1=Local; 2=Metastatic)
NORMAL SAMPLES
TISSUE_ID	GCI_DIAGNOSIS	TUMOR_PERCENT	WEIGHT (GRAMS)	age	sex	BMI	TOBACCO	TOBACCO_PERDAY	TOBACCO_TYPE	TOBACCO_USEYEARS	TOBACCO_CURRENT	TOBACCO_QUITYEARS	DRINK_ALCOHOL	DRINKS_NUMBER	CELL_TYPE	CANCERS	CANCER_YOU	CANCER_FATHER	CANCER_MOTHER	CANCER_ANYBROTHER	CANCER_ANYSISTER	CANCER_FATHERFATHER	CANCER_FATHERMOTHER	CANCER_MOTHERMOTHER	CANCER_MOTHERFATHER
1	NORMAL BREAST	0	1.77	49	FEMALE	23.6	NO	.		.		.	NO	.	FIBROCYSTIC CHANGE
2	NORMAL BREAST	0	1.52	50	FEMALE	20.8	NO	.		.		.	NO	.	CHRONIC INFLAMMATION
3	NORMAL BREAST	0	2.09	50	FEMALE	24.9	NO	.		.		.	NO	.	FIBROCYSTIC CHANGE
4	NORMAL BREAST	0	1.49	45	FEMALE	20.5	NO	.		.		.	NO	.	FIBROADENOMA
5	NORMAL BREAST	0	2.08	45	FEMALE	18.7	NO	.		.		.	NO	.	CHRONIC INFLAMMATION
6	NORMAL BREAST	0	1.08	31	FEMALE	31.5	NO	.		.		.	NO	.	FIBROCYSTIC CHANGES (APOCRINE METAPLASIA, APOCRINE CYSTS, INTRALOBULAR FIBROSIS)
7	NORMAL BREAST	0	0.43	50	FEMALE	24.3	YES	20	CIGARETTES	25	NO	3	YES	3	BENIGN FIBROCYSTIC CHANGES WITH MICROCALCIFICATION AND FOCI OF SCLEROSING ADENOSIS	BREAST CANCER	X				X			X
8	NORMAL BREAST	0	2.07	40	FEMALE	20.1	NO	.		.		.	NO	.	FIBROCYSTIC CHANGE
9	NORMAL BREAST	0	0.5	38	FEMALE	33.9	NO	.		.		.	NO	.	NORMAL BREAST TISSUE
10	NORMAL BREAST	0	0.85	35	FEMALE	40.9	NO	.		.		.	NO	.	MILD STROMAL CHRONIC INFLAMMATION, NORMAL BREAST TISSUE	PROSTATE CANCER						X
11	NORMAL BREAST	0	2.04	43	FEMALE	21.2	NO	.		.		.	NO	.	FIBROCYSTIC CHANGE
12	NORMAL BREAST	0	0.54	35	FEMALE	23.7	NO	.		.		.	NO	.	NORMAL BREAST TISSUE
13	NORMAL BREAST	0	2.2	35	FEMALE	19.9	NO	.		.		.	NO	.	FIBROCYSTIC CHANGE
14	NORMAL BREAST	0	0.87	30	FEMALE	29.8	NO	.		.		.	NO	.	NO SPECIFIC PATHOLOGIC CHANGE
15	NORMAL BREAST	0	1.16	32	FEMALE	38.7	NO	.		.		.	NO	.	NORMAL BREAST TISSUE	PROSTATE CANCER						X
16	NORMAL BREAST	0	2.06	45	FEMALE	20.3	NO	.		.		.	NO	.	FIBROCYSTIC CHANGE
17	NORMAL BREAST	0	1.92	49	FEMALE	21.8	NO	.		.		.	NO	.	FIBROCYSTIC CHANGE
18	NORMAL BREAST	0	1.87	45	FEMALE	27.1	NO	.		.		.	NO	.	BENIGN CHANGES NO HYPERPLASIA IDENTIFIED	RENAL CANCER							X
19	NORMAL BREAST	0	0.48	35	FEMALE	30.5	NO	.		.		.	NO	.	BENIGN BREAST TISSUE WITH FOCAL DENSE FIBROSIS
20	NORMAL BREAST	0	0.38	61	FEMALE	26.5	NO	.		.		.	NO	.	NORMAL BREAST TISSUE	LUNG CANCER				X
21	NORMAL BREAST	0	0.73	39	FEMALE	36.9	NO	.		.		.	NO	.	FOCAL EPITHELIAL HYPERPLASIA	BREAST CANCER	X
22	NORMAL BREAST	0	0.75	49	FEMALE	39.7	YES	15	CIGARETTES	30	YES	.	NO	.	FATTY BREAST TISSUE
23	NORMAL BREAST	0	0.33	48	FEMALE	34	NO	.		.		.	NO	.	NORMAL BREAST TISSUE
24	NORMAL BREAST	0	0.49	33	FEMALE	44.8	NO	.		.		.	NO	.	BENIGN SKIN AND MAMMARY TISSUE
25	NORMAL BREAST	0	0.73	57	FEMALE	28.7	NO	.		.		.	NO	.	FATTY BREAST TISSUE; FIBROCYSTIC MASTOPATHY	OVARIAN CANCER	X
26	BENIGN BREAST DISEASE	0	0.4	47	FEMALE	32.6	YES	10	CIGARETTES	5	YES	.	NO	.	FIBROCYSTIC DISEASE WITHOUT ATYPISM
27	BENIGN BREAST DISEASE	0	0.32	33	FEMALE	22.6	NO	.		.		.	NO	.	FIBROADENOMA/BENIGN FIBROCYSTIC CHANGES
28	NORMAL BREAST	0	0.61	33	FEMALE	36.7	NO	.		.		.	NO	.	FIBROCYSTIC CHANGE AND INTRADUCTAL CALCIFICATIONS
29	NORMAL BREAST	0	0.46	48	FEMALE	36.6	NO	.		.		.	NO	.	UNREMARKABLE SKIN AND BENIGN BREAST TISSUE AND HYALINIZED FIBROADENOMA
30	NORMAL BREAST	0	0.26	33	FEMALE	44.8	NO	.		.		.	NO	.	BENIGN SKIN AND MAMMARY TISSUE
31	BENIGN BREAST DISEASE	0	0.45	49	FEMALE	25	NO	.		.		.	NO	.	FIBROCYSTIC CHANGES WITH FOCAL APOCRINE METAPLASIA AND MICROCALCIFICATIONS
32	BENIGN BREAST DISEASE	0	0.43	37	FEMALE	28.2	NO	.		.		.	NO	.	BENIGN FIBROCYSTIC DISEASE	COLON CANCER			X
33	NORMAL BREAST	0	0.6	40	FEMALE	27.6	NO	.		.		.	NO	.	FIBROCYSTIC CHANGE (FIBROADIPOSE TISSUE)
34	NORMAL BREAST	0	0.57	40	FEMALE	37.3	NO	.		.		.	NO	.	PREDOMINANTLY FATTY BREAST TISSUE (NORMAL)
35	NORMAL BREAST	0	0.4	46	FEMALE	49	NO	.		.		.	NO	.	SKIN AND FATTY BREAST TISSUE (MACROMASTIA)
36	NORMAL BREAST	0	0.89	45	FEMALE	30	NO	.		.		.	NO	.	SKIN AND FATTY BREAST TISSUE WITH FIBROADENOMA	NONE OF THE ABOVE CANCER	X			X	X	X	X	X	X
37	NORMAL BREAST	0	0.38	57	FEMALE	20.9	YES	20	CIGARETTES	40	YES	.	NO	.	BREAST TISSUE WITH NO SPECIFIC PATHOLOGIC CHANGES	CERVICAL CANCER			X
38	NORMAL BREAST	0	0.58	54	FEMALE	40	NO	.		.		.	NO	.	FATTY ATROPHIC BREAST TISSUE/HYALINIZED AND CALCIFIED FIBROADENOMA	COLON CANCER		X
39	NORMAL BREAST	0	0.43	52	FEMALE	24.8	NO	.		.		.	NO	.	FIBROCYSTIC CHANGES WITH DUCT ECTASIA, APOCRINE METAPLASIA, ATROPHY AND STROMAL FIBROSIS
40	NORMAL BREAST	0	0.45	41	FEMALE	26.3	YES	25	CIGARETTES	23	YES	.	NO	.	PREDOMINATELY FATTY BREAST TISSUE, FOCAL DUCTAL ECTASIA WITH APOCRINE METAPLASIA
41	NORMAL BREAST	0	0.32	61	FEMALE	26.5	NO	.		.		.	NO	.	NORMAL BREAST TISSUE	LUNG CANCER				X
42	NORMAL BREAST	0	0.49	49	FEMALE	33.6	NO	.		.		.	NO	.	BENIGN BREAST TISSUE (CLINICAL HYPERTROPHY)	BREAST CANCER			X
43	NORMAL BREAST	0	0.25	36	FEMALE	28.9	NO	.		.		.	NO	.	NORMAL BREAST TISSUE
44	NORMAL BREAST	0	1.17	50	FEMALE	26.6	NO	.		.		.	YES	.	BENIGN BREAST TISSUE (MAMMARY HYPERPLASIA)
45	NORMAL BREAST	0	0.28	44	FEMALE	23.8	YES	20	CIGARETTES	20	NO	.	NO	.	INTERLOBULAR AND PERILOBULAR FIBROSIS AND FOCAL ADENOSIS WITH SLIGHT EPITHELIAL HYPERPLASIA	CERVICAL CANCER	X							X
46	NORMAL BREAST	0	0.37	32	FEMALE	35.3	NO	.		.		.	NO	.	NORMAL BREAST TISSUE CONSISTENT WITH MACROMASTIA AND FIBROCYSTIC CHANGE	LEUKEMIA			X
47	NORMAL BREAST	0	0.35	39	FEMALE	46.9	YES	20	CIGARETTES	15	NO	6	YES	2	NORMAL BREAST TISSUE
48	NORMAL BREAST	0	1.16	38	FEMALE	29.8	YES	20	CIGARETTES	20	NO	.	YES	4	BENIGN BREAST TISSUE CONSISTENT WITH MAMMARY HYPERPLASIA WITH ASSOCIATED MICROCALCIFICATIONS	OTHER GI CANCER		X
49	NORMAL BREAST	0	0.84	56	FEMALE	25.4	YES	5	CIGARETTES	17	NO	19	NO	.	BENIGN BREAST TISSUE WITH MAMMARY HYPERPLASIA	COLON CANCER			X
50	NORMAL BREAST	0	0.42	39	FEMALE	34.1	YES	20	CIGARETTES	10	NO	0	YES	3	NORMAL BREAST TISSUE
51	NORMAL BREAST	0	0.62	38	FEMALE	42.2	YES	20	CIGARETTES	17	NO	9	NO	.	NORMAL BREAST TISSUE	LUNG CANCER		X
52	NORMAL BREAST	0	0.41	53	FEMALE	40.8	NO	.		.		.	NO	.	NORMAL BREAST TISSUE	BREAST CANCER			X		X
53	NORMAL BREAST	0	0.31	38	FEMALE	33.9	NO	.		.		.	NO	.	NORMAL BREAST TISSUE
54	NORMAL BREAST	0	0.37	33	FEMALE	34.9	NO	.		.		.	NO	.	NORMAL BREAST TISSUE
55	NORMAL BREAST	0	0.55	38	FEMALE	29.8	NO	.		.		.	NO	.	NORMAL BREAST TISSUE
56	NORMAL BREAST	0	0.5	64	FEMALE	29	NO	.		.		.	NO	.	BENIGN BREAST TISSUE	PROSTATE CANCER		X
57	NORMAL BREAST	0	0.32	37	FEMALE	28.3	NO	.		.		.	NO	.	MILD CHRONIC CERVICITIS WITH SQUAMOUS METAPLASIA BENIGN OVARIES W/HEMORRHAGIC CORPUS LUTEM AND FOLLICLE CYST
58	NORMAL BREAST	0	1.5	37	FEMALE	22.9	NO	.		.		.	NO	.	LEFT BREAST: FIBROCYSTIC WITH FOCI OF FLORID ADENOSIS AND INTRADUCTAL HYPERPLASIA RT BREAST: FIBROCYSTIC MASTOPATHY WITH FOCI OF ADENOSIS AND SCLEROSING ADENOSIS	BREAST CANCER	X						X
59	NORMAL BREAST	0	1.36	58	FEMALE	44.3	NO	.		.		.	NO	.	BENIGN BREAST TISSUE (NO CA TISSUE AVAILABLE FOR GCI)	BREAST CANCER	X
60	NORMAL BREAST	0	0.41	41	FEMALE	42.6	NO	.		.		.	NO	.	BENIGN BREAST TISSUE
61	NORMAL BREAST	0	1.03	63	FEMALE	20.2	YES	5	CIGARETTES	12	NO	10	YES	1	FIBROCYSTIC MASTOPATHY WITH FOCAL MICROCALCIFICATIONS	BREAST CANCER	X		X
62	NORMAL BREAST	0	1.37	45	FEMALE	27.1	NO	.		.		.	NO	.	BENIGN CHANGES NO HYPERPLASIA IDENTIFIED	RENAL CANCER							X
63	NORMAL BREAST	0	0.67	41	FEMALE	34	YES	40	CIGARETTES	25	YES	.	NO	.	BENIGN BREAST TISSUE, FIBORCYSTIC CHANGE WITH FLORID DUCTAL HYPERPLASIA, SCLEROSING ADENOSIS, MICROCYST FORMATION AND FOCAL MICROCALCIFCATIONS	BREAST CANCER					X
64	NORMAL BREAST	0	0.46	61	FEMALE	30.7	NO	.		.		.	NO	.	FIBROCYSTIC CHANGES WITHOUT ATYPIA	BREAST CANCER	X
65	NORMAL BREAST	0	1.35	47	FEMALE	34.3	YES	10	CIGARETTES	32	YES	.		.	BENIGN BREAST
66	BENIGN BREAST DISEASE	0	1.51	64	FEMALE	24.7	YES	40	CIGARETTES	34	NO	6	YES	21	BENIGN BREAST TISSUE DEMONSTRATING FIBROCYSTIC MASTOPATHY WITH ASSOCIATED CYST FORMATION
67	NORMAL BREAST	0	0.79	34	FEMALE	43.2	NO	.		.		.	NO	.	NORMAL BREAST TISSUE	BREAST CANCER			X
68	NORMAL BREAST	0	0.65	49	FEMALE	39.7	YES	15	CIGARETTES	30	YES	.	NO	.	FATTY BREAST TISSUE
69	BENIGN BREAST DISEASE	0	0.71	44	FEMALE	27.4	YES	20	CIGARETTES	16	YES	.	YES	3	FIBROCYSTIC CHANGES/APOCRINE METAPLASIA	BREAST CANCER	X
70	BENIGN BREAST DISEASE	0	0.76	49	FEMALE	31.4	YES	10	CIGARETTES	20	YES	.	NO	.	NORMAL BREAST TISSUE	COLON CANCER				X
71	BENIGN BREAST DISEASE	0	0.25	45	FEMALE	32.8	NO	.		.		.	YES	4	FIBROADENOMA
72	BENIGN BREAST DISEASE	0	0.87	42	FEMALE	26.1	NO	.		.		.	NO	.	NORMAL BREAST TISSUE	LUNG CANCER		X
73	BENIGN BREAST DISEASE	0	0.35	44	FEMALE	26.5	NO	.		.		.	NO	.	NORMAL BREAST
74	NORMAL BREAST	0	0.32	56	FEMALE	31.6	NO	.		.		.	NO	.	NORMAL BREAST TISSUE	COLON CANCER		X
75	BENIGN BREAST DISEASE	0	0.76	34	FEMALE	25.6	NO	.		.		.	NO	.	NORMAL BREAST TISSUE	SKIN CANCER		X
76	NORMAL BREAST	0	0.77	47	FEMALE	26	NO	.		.		.	NO	.	NORMAL TISSUE	CERVICAL CANCER			X
77	BENIGN BREAST DISEASE	0	0.6	31	FEMALE	44.8	NO	.		.		.	NO	.	NORMAL BREAST TISSUE
78	BENIGN BREAST DISEASE	0	0.87	35	FEMALE	29.2	NO	.		.		.	YES	.	NORMAL SKIN AND BREAST TISSUE	LUNG CANCER							X
79	NORMAL BREAST	0	1.16	32	FEMALE	38.7	NO	.		.		.	NO	.	NORMAL BREAST TISSUE	PROSTATE CANCER						X
80	BENIGN BREAST DISEASE	0	1.17	38	FEMALE	48.7	YES	5	CIGARETTES	2	NO	20	YES	2	NORMAL BREAST TISSUE	LUNG CANCER									X
81	BENIGN BREAST DISEASE	0	0.41	34	FEMALE	34.3	NO	.		.		.	NO	.	NORMAL BREAST TISSUE	NONE OF THE ABOVE CANCER	X	X	X	X		X	X	X	X
82	BENIGN BREAST DISEASE	0	0.42	46	FEMALE	30.4	NO	.		.		.	NO	.	NORMAL BREAST TISSUE
83	BENIGN BREAST DISEASE	0	0.56	32	FEMALE	23.9	YES	3	CIGARETTES	10	NO	.	YES	.	SKIN AND NORMAL BREAST TISSUE
84	BENIGN BREAST DISEASE	0	0.77	33	FEMALE	33.3	NO	.		.		.	YES	.	NORMAL - BENIGN BREAST TISSUE
85	BENIGN BREAST DISEASE	0	0.79	35	FEMALE	41.7	NO	.		.		.	YES	4	BENIGN HAMARTOMA	PROSTATE CANCER		X
86	BENIGN BREAST DISEASE	0	0.3	49	FEMALE	22.9	YES	10	CIGARETTES	3	NO	15	YES	1	NORMAL BREAST/LIPOMA UNK
87	NORMAL BREAST	0	0.43	32	FEMALE	24.5		.		.		.		.	NO HISTOLOGIC ABNORMALITY
88	BENIGN BREAST DISEASE	0	0.6	37	FEMALE	28.2	NO	.		.		.	NO	.	NORMAL BREAST TISSUE
89	NORMAL BREAST	0	0.89	35	FEMALE	23.7	NO	.		.		.	NO	.	NORMAL BREAST TISSUE
90	BENIGN BREAST DISEASE	0	0.38	58	FEMALE	28.7	NO	.		.		.	NO	.	FIBROCYSTIC DISEASE	NONE OF THE ABOVE CANCER	X	X	X
91	BENIGN BREAST DISEASE	0	0.38	45	FEMALE	33.1	YES	10	CIGARETTES	15	NO	.	YES	4	FIBROEPITHELIAL NEOPLASM - BENIGN	NONE OF THE ABOVE CANCER	X	X	X	X	X	X	X		X
92	NORMAL BREAST	0	1.24	56	FEMALE	29.7	NO	.		.		.	YES	2	BENIGN BREAST TISSUE WITH CLINICAL MAMMARY HYPERTROPHY
93	NORMAL BREAST	0	0.72	56	FEMALE	39.9	NO	.		.		.	NO	.	FATTY BREAST TISSUE WITH DUCTAL ECTASIA	BREAST CANCER			X
94	BENIGN BREAST DISEASE	0	0.5	50	FEMALE	31.3	NO	.		.		.	NO	.	NORMAL BREAST TISSUE
95	NORMAL BREAST	0	0.85	31	FEMALE	28.2	YES	2	CIGARETTES	.	YES	.	YES	2	NO SIGNIFICANT HISTOPATHOLOGY	SKIN CANCER									X
96	NORMAL BREAST	0	0.41	43	FEMALE	31.6	NO	.		.		.	YES	1	NORMAL BREAST TISSUE/BENIGN BREAST GLANDULAR ELEMENTS	NONE OF THE ABOVE CANCER	X		X	X	X	X	X	X	X
97	NORMAL BREAST	0	0.51	33	FEMALE	26.6	NO	.		.		.	NO	.	NORMAL BREAST TISSUE
98	NORMAL BREAST	0	0.66	41	FEMALE	19.7	YES	20	CIGARETTES	14	NO	14	YES	2	FIBROADENOMA
99	NORMAL BREAST	0	0.37	44	FEMALE	26.6	YES	60	CIGARETTES	20	NO	9	YES	.	FOCAL FIBROADENOMATOID HYPERPLASIA AND MODERATE NON-ATYPICAL EPITHELIAL HYPERPLASIA WITH FOCAL MICROCALCIFICATION	NONE OF THE ABOVE CANCER	X	X	X		X
100	NORMAL BREAST	0	0.53	49	FEMALE	36.9	NO	.		.		.	NO	.	CYSTIC DUCTS AND INTRALOBULAR FIBROSIS	COLON CANCER								X
101	NORMAL BREAST	0	0.56	31	FEMALE	23	YES	5	CHEWING TOBACCO (PINCHES)	5	YES	.	YES	1	NORMAL BREAST WITH FIBROADENOMA
102	NORMAL BREAST	0	0.6	61	FEMALE	32.6	YES	5	CIGARETTES	10	NO	15	YES	1	BENIGN BREAST TISSUE W/FOCAL FIBROCYSTIC CHANGE	PROSTATE CANCER		X
103	BENIGN BREAST DISEASE	0	0.53	65	FEMALE	29.9	YES	60	CIGARETTES	25	NO	13	NO	.	PROLIFERATIVE FIBROCYSTIC CHANGES WITH MICROCALCIFICATIONS	BREAST CANCER	X							X
104	NORMAL BREAST	0	0.33	39	FEMALE	26.4	NO	.				.		.

## Sheet: sheet 2
